# Intakes of Added Sugars, with a Focus on Beverages and the Associations with Nutrient Adequacy in US Adults (NHANES 2003–2018)

**DOI:** 10.3390/nu15183916

**Published:** 2023-09-09

**Authors:** Laurie Ricciuto, Victor L. Fulgoni, P. Courtney Gaine, Maria O. Scott, Loretta DiFrancesco

**Affiliations:** 1 Department of Nutritional Sciences, University of Toronto, Toronto, ON M5S 1A1, Canada; laurie.ricciuto@utoronto.ca; 2 Nutrition Impact, LLC, Battle Creek, MI 49014, USA; vic3rd@aol.com; 3 The Sugar Association, Inc., Washington, DC 20005, USA; gaine@sugar.org (P.C.G.); mscott@sugar.org (M.O.S.); 4 Source! Nutrition, Toronto, ON M6S 5A6, Canada

**Keywords:** added sugars, sweetened beverages, nutrient intake, nutrient adequacy, adults, US, NHANES

## Abstract

The Dietary Guidelines for Americans recommend adults increase their intake of nutrients that are under-consumed while limiting their intake of added sugars, sodium, and saturated fats. The purpose of this study was to examine the relationship between added sugars intake from specific types of beverages with added sugars (soft drinks, fruit drinks, sports and energy drinks, coffee and tea, and flavored milk) and nutrient adequacy among US adults (19+ y). Data from eight consecutive 2-y cycles of NHANES were combined (2003–2004 through 2017–2018), and regression analysis was conducted to test for trends in quantiles of added sugars intake from each beverage source and the rest of the diet (excluding those beverages) and nutrient adequacy. Results revealed significant associations that varied in direction according to the added sugars source, negative for some (i.e., soft drinks) in terms of greater percentages of adults not meeting a defined threshold of nutrient adequacy with higher added sugars intakes, and positive for others (i.e., fruit drinks, flavored milk, the rest of the diet) in terms of lower percentages of adults not meeting nutrient thresholds. In conclusion, the contribution of different added sugars sources to nutrient intakes is a critical consideration in developing population-based dietary recommendations.

## 1. Introduction

The 2020–2025 Dietary Guidelines for Americans (DGA) provide recommendations on dietary intake patterns for the US population based on current dietary intakes and the scientific evidence on diet-related chronic disease. For adults, reducing the risk of diet-related chronic disease is a key consideration in the recommendations, as more than one-half of US adults are living with at least one chronic disease; older adults (≥60 y), in particular, are at higher risk of chronic disease and other health conditions related to the aging process [1]. As a result, the DGA recommends adults increase their intake of nutrients that are under-consumed (e.g., calcium and vitamin D as related to osteoporosis) while reducing their intake of added sugars, sodium, and saturated fats. For added sugars, the DGA recommends limiting intake to ≤10% energy per day by limiting food and beverages high in calories from added sugars [1]. Such a blanket recommendation for added sugars may be problematic and result in unintended consequences because it does not consider the various sources of added sugars in the diet, which vary in both added sugars and essential nutrient content [2,3]; thus, any changes in the consumption of these sources may not only impact added sugars intake, but also nutrient intakes.

This concept of reducing added sugars intake without compromising nutrient intake is illustrated in the area of sweetened beverages. Sweetened beverages are the top source of added sugars in the US diet [3], and thus, reducing their consumption in order to reduce added sugars intake is a main emphasis in the DGA [1]. An analysis of the National Health and Nutrition Examination Survey (NHANES) 2017–2018 data have shown that beverages as a whole (water, alcoholic beverages, coffee/tea, sweetened beverages, milk and 100% juice) contribute 54% of added sugars intake, and also contribute meaningfully to intakes of protein (7%) and calcium, magnesium, phosphorus, potassium and vitamins C and D (14–38%) [4]. Furthermore, sweetened beverages, in particular, are typically examined as an aggregate group, yet they vary in their nutritional composition; for example, soft drinks provide no essential nutrients, and fruit drinks provide vitamin C [2]. Sweetened beverages are also typically examined in isolation; however, considering them in the context of the total diet would provide a more complete assessment.

The purpose of our study was to examine the relationship between added sugars intake from specific types of beverages with added sugars (soft drinks, fruit drinks, sports and energy drinks, coffee and tea, and flavored milk) and nutrient adequacy for public health concerns and nutrients that are under-consumed among US adults; and, for context, to examine the relationship between added sugars intake from the rest of the diet (excluding beverages with added sugars) and nutrient adequacy.

## 2. Materials and Methods

### 2.1. Data

Diet and health are monitored regularly in the US through NHANES, a nationally representative cross-sectional survey of non-institutionalized civilian residents conducted by the US National Center for Health Statistics (NCHS), which is part of the Centers for Disease Control and Prevention (CDC). Written, informed consent is obtained from all participants. Details on the NHANES survey design, data collection, and analytic procedures are reported elsewhere [5,6]. Data from eight consecutive 2-y cycles of NHANES (those with two 24 h dietary recalls) were combined (2003–2004 through 2017–2018) in order to provide sufficient sample size to examine intakes of specific types of beverages with added sugars. Only subjects with dietary intake data from two non-consecutive 24 h recalls were included in the analyses to enable the calculation of usual intakes (UI) required for the assessment of nutrient adequacy. Of those age 19 + y, n = 10,075 were excluded due to missing or unreliable dietary data and n = 1053 pregnant or lactating females were also excluded, resulting in a final analytic sample size of n= 35,128, separated into three age groups: n = 18,110 age 19–50 y; n = 11,379 age 51–70 y; and, n = 5639 age 71+ y (Supplemental Figure S1). These age groups were selected because they correspond with those in the Dietary Reference Intakes (DRI), and thus reflect the nutrient requirements across these life stages [7].

### 2.2. Added Sugars Intake

The USDA Food Patterns and Equivalents Database (FPED) converts food and beverage intakes to food group equivalents corresponding to those in the DGA [8]. The added sugars group is composed of caloric sweeteners defined as “sugars that are added to foods as an ingredient during preparation, processing or at the table; and does not include naturally occurring sugars, such as lactose present in milk and fructose present in whole or cut fruit and 100% fruit juice” [8]. Added sugars values are provided as teaspoon equivalents in the FPED and reflect added sugars intakes from all foods and beverages; values were converted to grams and kilocalories (4.2 g/tsp and 4.0 kcal/g, respectively). The beverages with added sugars we analyzed were based on the 2017–2018 What We Eat in America (WWEIA) food categories, in which foods and beverages are grouped by the USDA based on similar nutrient content and common use in the diet [9]. The WWEIA food categories include individual categories for soft drinks, fruit drinks, sports and energy drinks, coffee and tea, and flavored milk, and an aggregated “sweetened beverages” category, which combines soft drinks, fruit drinks, sport, and energy drinks, and also includes nutritional beverages and smoothies and grain drinks (Table 1). Mean added sugars intake from each of these beverage categories was determined using the FPED specific to each NHANES cycle and the average of both dietary recalls. For context, we also calculated mean added sugars intake from the rest of the diet, which was composed of all foods and beverages except the beverages with added sugars that we examined (i.e., the WWEIA sweetened beverage group, coffee and tea, and flavored milk).

### 2.3. Added Sugars Intake and Nutrient Adequacy

To account for differences in energy intake over time, added sugars intake was calculated as a percentage of total daily calories (% kcal). Mean added sugars intake within each beverage group was divided into quantiles; non-consumers comprised the first quantile, and then consumers were divided into tertiles (quantiles 2, 3, and 4). Given the very low number of non-consumers (<0.5% of the population) for the rest of the diet, added sugars intake from the rest of the diet was divided into quartiles.

Intakes of ten nutrients were obtained from NHANES dietary intake files. They were: those of public health concern among US adults (calcium, potassium, vitamin D, and dietary fiber) [1]; those more likely to be under-consumed by older adults (protein and vitamin B12) [1]; and those with ≥50% of intakes below the estimated average requirement (EAR) for at least one age/sex group (magnesium and vitamins A, C, and E) [10]. To estimate UI and distribution of intakes of nutrients for each age group, the National Cancer Institute (NCI) method was used [11]. Given most nutrients were consumed on most days by most subjects, the one-part model was used for the UI estimations. The 2 d of intake, using 2-d sampling weights, was used to obtain percentiles of intake and necessary variance estimates. Covariates used in the NCI UI estimations were a day of the week of the 24 h recall (coded as weekend (Friday to Sunday) or weekday (Monday to Thursday)) and sequence of dietary recall (first or second). Balanced repeated replication was performed to generate standard errors; balanced repeated replication weights were generated using Fay adjustment factor M = 0.3 with perturbation factor of 0.7, which were then adjusted to match initial sample weights within age, sex and race and ethnicity groups.

Nutrient adequacy was assessed using the EAR cut-point method [7], which estimates the percentage of an age group with intakes below requirements. There are no EARs established for potassium and dietary fiber, and thus, assessment for those nutrients was limited to a determination of the percentage of individuals with intakes greater than the adequate intake (AI). All percentages were calculated within each quantile of added sugars intake for each beverage source and for the rest of the diet. Nutrient intakes from dietary supplements were not included because the primary research question focused on intakes from beverages with added sugars and from the rest of the diet.

### 2.4. Statistical Analyses

Data were analyzed using SAS 9.4 (SAS Institute, Cary, NC, USA). Dietary sample weights, primary sampling units, and strata provided by NHANES were used to adjust for the complex survey sampling design, design changes across survey cycles, non-response rates, and oversampling of certain subgroups. While varying for each cycle of NHANES, the interview response rate was approximately 50% for adults in NHANES 2017–2018 [12]. Regression analysis was used to examine associations between quantiles of added sugars intake from beverages with added sugars and from the rest of the diet and % <EAR (or % >AI) for selected nutrients. Within each beverage source, linear trends in % <EAR (or % >AI) across quantiles of added sugars intake (quantiles 1–4) were assessed. Additionally, the difference in % <EAR (or % >AI) between non-consumers (quantile 1) and consumers (quantiles 2, 3, and 4) was assessed, and given the large number of non-consumers for many of the beverage sources, the linear trends across tertiles of consumers only (quantiles 2–4) were also assessed. Furthermore, for beverages in which changes in consumption occurred over time (soft drinks, fruit drinks, sports and energy drinks, coffee and tea), trend analyses were repeated on 4-y cycles for the combined age group (19+ y) for sufficient sample size. The analyses were also stratified by sex; however, as there were few differences in males and females, and to simplify the presentation of the results, only sex-combined results are reported. A value of *p* < 0.01 was deemed statistically significant; however, differences that were *p* < 0.05 and had an effect size considered nutritionally significant (a difference of 5% units <EAR or >AI) were also highlighted.

## 3. Results

### 3.1. Demographics

The average age of the population examined was 47.3 ± 0.2 y, with 48.8 ± 0.36% male and 51.2 ± 0.2% female. Approximately 29% of the population was of normal weight, and about 33% and 37% were overweight or obese, respectively. Approximately 15% of the population had less than a high school education, while about 24% had a high school diploma or equivalent, and about 60% of the population had some college or a Bachelor’s degree (Supplemental Table S1).

### 3.2. Consumption of Beverage Sources of Added Sugars and Added Sugars Intake and Patterns over Time

The percentage of adults who reported consuming any of the beverages with added sugars in the previous 24 h (i.e., consumers) decreased with increasing age, with the exception of flavored milk (Table 2). Soft drinks had the highest levels of reporting, at 48.8%, among those aged 19–50 y, which was twice the level of reporting compared to those aged 71+ y (at 22.0%). The second highest levels of reporting were for fruit drinks and coffee and tea, at 23.2% and 21.4%, respectively, among those aged 19–50 y, 16.6% and 16.7% among those aged 51–70 y, and 16.6% and 13.3% among those age 71+ y.

Mean daily energy intake was 2215 ± 10 kcal among those aged 19–50 y, with 323 ± 4 kcal coming from added sugars; 2012 ± 12 kcal among those aged 51–70, with 254 ± 3 kcal from added sugars; and 1746 ± 14 kcal among those age 71+ y, with 210 ± 4 kcal from added sugars. Added sugars (% kcal) from all beverage sources combined accounted for approximately 52%, 36%, and 25% of total added sugars intake among those aged 19–50 y, 51–70 y, and 71+ y, respectively, and added sugars intake from the rest of the diet accounted for 48%, 64% and 75% of total added sugars intake (Table 3). Among all individuals, added sugars intake from all beverage sources except flavored milk declined with age, while the opposite was observed for added sugars intake from the rest of the diet, with mean added sugars intake increasing with age. Among consumers only, there were declines in added sugars intake from soft drinks and fruit drinks with increasing age, at 9.3% kcal and 5.0% kcal, respectively, among those aged 19–50 y, and 6.3% kcal and 4.0% kcal, respectively, among those age 71+ y; whereas added sugars intakes from sports and energy drinks and coffee and tea among consumers only were similar among the age groups.

**Figure 1:**
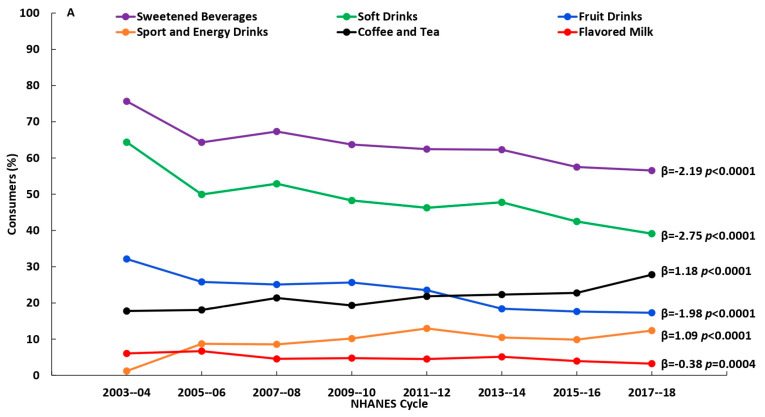

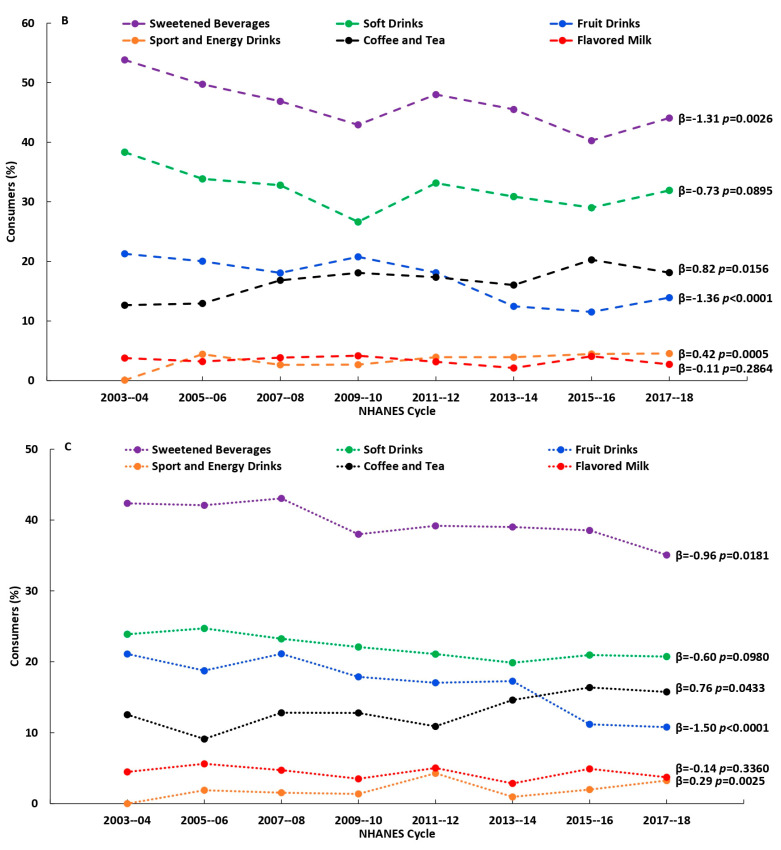
Percent of adults reporting intake (consumers) of beverage sources of added sugars by NHANES cycle among (**A**) 19–50 y (n = 18,110), (**B**) 51–70 y (n = 11,379) and (**C**) 71+ y (n = 5639); β and *p*-values from linear trend analysis, significant at *p* < 0.01; source NHANES 2003–2004 to 2017–2018.

**Figure 2:**
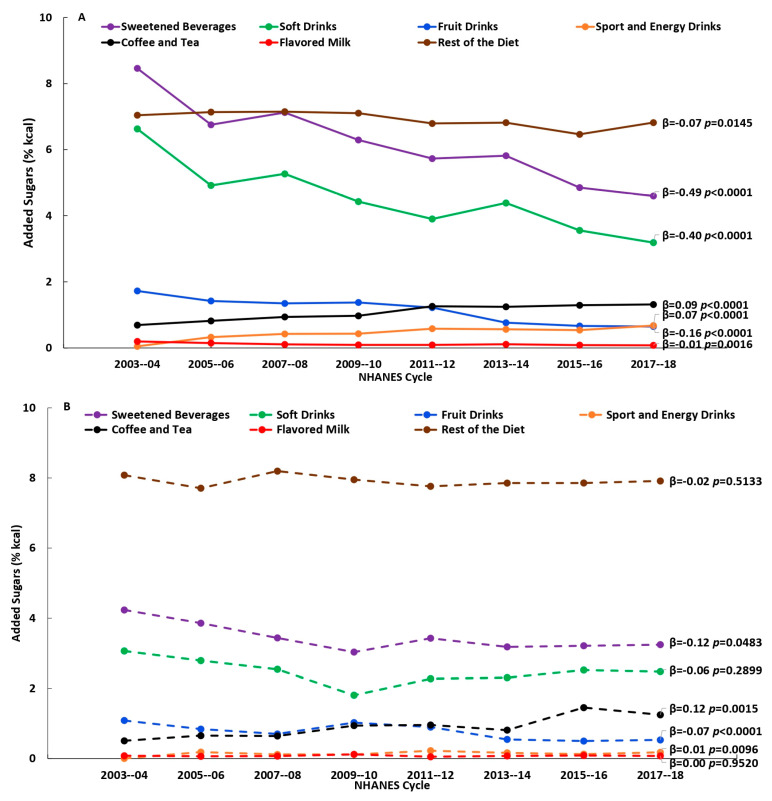

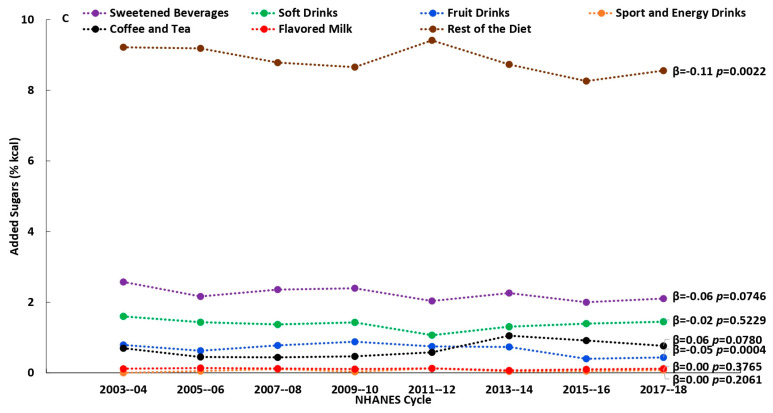
Added sugars intake (% kcal) from beverage sources and the rest of the diet by NHANES cycle among all individuals, (**A**) 19–50 y, (**B**) 51–70 y and (**C**) 71+ y; β and *p*-values from linear trend analysis, significant at *p* < 0.01; source NHANES 2003–2004 to 2017–2018.

**Figure 3:**
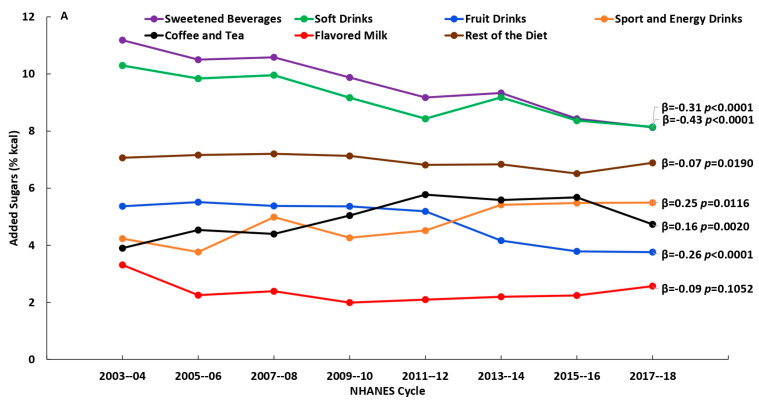

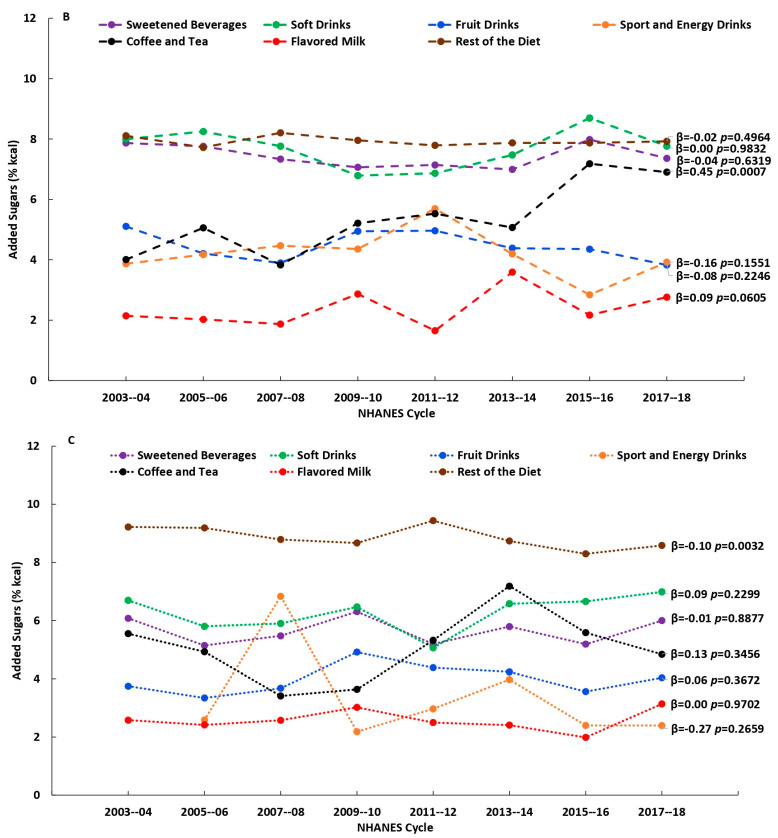
Added sugars intake (% kcal) from beverage sources and the rest of the diet by NHANES cycle among consumers, (**A**) 19–50 y, (**B**) 51–70 y and (**C**) 71+ y; β and *p*-values from linear trend analysis, significant at *p* < 0.01; source NHANES 2003–2004 to 2017–2018.

There were significant changes (*p* < 0.01) in the consumption of beverage sources and in added sugars intake from the beverage sources over the 16-year time span (2003–2018), to varying degrees depending on age. Among younger adults (19–50 y), there were decreases in the percentages who reported consuming soft drinks, fruit drinks, and flavored milk and increases in the percentages who reported consuming sports and energy drinks and coffee and tea; among the older adult groups (51–70 y and 71+ y) there were decreases in percentages who reported consuming fruit drinks and increases in percentages who reported consuming sports and energy drinks (Figure 1A–C and Supplemental Table S2). Added sugars intake (% kcal) from the beverages sources generally were in the same direction over time: among younger adults (19–50 y) added sugars intake from soft drinks, fruit drinks and flavored milk decreased, while added sugars intake from sports and energy drinks and coffee and tea increased; among those age 51–70 y, added sugars intake from fruit drinks decreased, while added sugars intake from sports and energy drinks and coffee and tea increased; and, among those age 71+ y, added sugars intake from fruit drinks decreased (Figure 2A–C and Supplemental Table S3). Among consumers only, there were decreases in added sugars intake from soft drinks and fruit drinks among those aged 19–50 y, and increases in added sugars intake from coffee and tea among those aged 19–50 y and 51–70 y, while there were no changes observed among consumers age 71+ y (Figure 3A–C and Supplemental Table S3). Results using added sugars intake expressed in grams are also provided in Supplemental Table S4.

### 3.3. Associations between Added Sugars Intake and Nutrient Adequacy by Beverage Source

Among consumers of beverages, tertile ranges of added sugars intake varied by beverage type and age (except flavored milk, with tertile ranges similar across age groups) (Table 4). The lowest added sugars intakes were from flavored milk and sports and energy drinks, with a range across tertiles of 1.4–2.4% and 2.5–4.9%, respectively, among those aged 19–50 y. The highest added sugars intakes were from soft drinks with a range across tertiles of 4.8–10.0% among those aged 19–50 y, followed by fruit drinks and coffee and tea at 2.4–5.2% and 2.4–5.1%, respectively, among those age 19–50 y.

Age 19–50 y: Significant relationships between added sugars intake and nutrient adequacy emerged for all beverage types (Table 5). For soft drinks, higher added sugars intake was associated with greater percentages of individuals with intakes below the EAR for magnesium (8.7% unit increase for every increase in quantile of added sugars, *p* < 0.05) and vitamins A and C (8.5% and 7.9% unit increases, respectively, *p* < 0.05), and a lower percentage above the AI for potassium (5% unit decrease across quantiles, *p* < 0.05). Additionally, among consumers of soft drinks, the percentages with intakes below the EAR for vitamins A and E were higher (19.8% and 11.1% units, respectively, *p* < 0.05), and the percentage with intakes above the AI for potassium was lower (11.7% units, *p* < 0.05) compared to non-consumers. In contrast, for fruit drinks, the percentage of individuals with intakes below the EAR for vitamin C decreased across quantiles (20.4% unit decrease with every increase in quantile of added sugars, *p* < 0.05); and for consumers, the percentage with intakes below the EAR for vitamin C was lower (45.8% units, *p* < 0.05) versus non-consumers of fruit drinks. For sports and energy drinks, the percentages of individuals with intakes below the EAR for calcium and vitamin B12 were lower among consumers of these beverages (9.7% and 4.5% units, respectively, *p* < 0.01) compared to non-consumers, while the percentage with intakes below the EAR for vitamin A was higher (8.0% units, *p* < 0.05) and the percentage with dietary fiber intakes above the AI was lower (2.0% units, *p* < 0.01). For coffee and tea, the percentages of individuals with intakes below the EAR for vitamins D and E increased across quantiles of added sugars but with very small effect sizes (0.6% and 1.0% unit increases, respectively, *p* < 0.01). For flavored milk, the percentages of individuals with intakes below the EAR for calcium, magnesium, and vitamin A decreased, and the percentage with potassium intakes above the AI increased across quantiles, with 11.9%, 9.8%, and 10.5% unit decrease (*p* < 0.01) and a 10.5% unit increase (*p* < 0.01), respectively, for every increase in quantile of added sugars. Additionally, graded relationships were observed among consumers of flavored milk; higher added sugars intake was associated with a lower percentage below the EAR for magnesium (9.1% unit decrease across tertiles of added sugars, *p* < 0.05) and a higher percentage above the AI for potassium (12.5% unit increase, *p* < 0.05).

51–70 y: Significant relationships between added sugars intake and nutrient adequacy emerged for all beverage types except coffee and tea (Table 6). For soft drinks, higher added sugars intake was associated with greater percentages of individuals with intakes below the EAR for magnesium (9.2% unit increase for every increase in quantile of added sugars, *p* < 0.01) and vitamins A and C (6.5% and 6.7% unit increases, respectively, *p* < 0.05) and a lower percentage of individuals with intakes above the AI for potassium (6.9% unit decrease, *p* < 0.05). Additionally, among consumers of soft drinks, the percentages with intakes below the EAR for vitamin E were higher (9.9% units, *p* < 0.05), and with intakes above the AI for dietary fiber was lower (10.6% units, *p* < 0.05) compared to non-consumers. In contrast, for fruit drinks, the percentage of individuals with intakes below the EAR for vitamin C decreased by 18.5% units (*p* < 0.05) for every increase in the quantile of added sugars, and among consumers, the percentage below the EAR for vitamin C was lower (42.3% units, *p* < 0.05) versus non-consumers of fruit drinks. Likewise, for sports and energy drinks, the percentage of individuals with intakes below the EAR for calcium decreased by 7.9% units (*p* < 0.05) for every increase in quantile of added sugars; and among consumers, the percentages with intakes below the EAR for calcium and vitamins B12 and E were lower by 17.4% units (*p* < 0.05), 4.6% units (*p* < 0.01) and 6.2% units (*p* < 0.01), respectively, versus non-consumers of these beverages. Similarly, for flavored milk, the percentage of individuals with intakes below the EAR for calcium decreased by 11.4% units (*p* < 0.05) for every increase in quantile of added sugars, and the percentage with intakes above the AI for potassium increased by 7.5% units (*p* < 0.05).

Age 71+ y: Significant relationships between added sugars intake and nutrient adequacy emerged for some beverage types (Table 7). For soft drinks, the percentages of individuals with intakes below the EAR for calcium and vitamin A increased by 4.9% and 7.7% unit increases (*p* < 0.05), respectively, with every increase in the quantile of added sugars. Additionally, for soft drinks, the percentages with intakes below the EAR for calcium, magnesium and vitamins A and E were higher among consumers by 9.9% units (*p* < 0.05), 17.8% units (*p* < 0.01), 15.4% units (*p* < 0.05) and 9.4% units (*p* < 0.01), respectively, versus non-consumers, and the percentages with potassium and dietary fiber intakes above the AI were lower by 14.6 units (*p* < 0.01) and 9.1% units (*p* < 0.05), respectively. In contrast, for fruit drinks, the percentage of individuals with intakes below the EAR for vitamin C decreased by 16.7% units (*p* < 0.05) with every increase in quantile of added sugars; and among consumers, the percentage was lower (34.3% units, *p* < 0.05) compared to non-consumers of fruit drinks. Similarly, for flavored milk, the percentages of individuals with intakes below the EAR for calcium and vitamin A decreased by 11.5% units (*p* < 0.05) and 4.5% units (*p* < 0.01), respectively, with every increase in quantile of added sugars. Additionally, among consumers of flavored milk, there was a graded relationship for magnesium, with a 17.3% unit decrease (*p* < 0.05) in the percentage of individuals with intakes below the EAR with every increase in tertile of added sugars. No relationships were observed for sports and energy drinks or for coffee and tea.

4-y cycle versus pooled sample analyses: Results from the 4-y cycle analysis on soft drinks, fruit drinks, sports and energy drinks, and coffee and tea among all adults (19+ y) were consistent with those from the pooled sample analysis, with associations occurring in the same directions (Supplemental Figure S2A–D, Supplemental Table S5).

### 3.4. Associations between Added Sugars from the Rest of the Diet and Nutrient Adequacy

Significant associations between added sugars from the rest of the diet and nutrient adequacy were observed for calcium, magnesium, potassium, and vitamins A, C, and D, with variations by age (Supplemental Table S6). Among all age groups, higher added sugars intake was associated with lower percentages of individuals with intakes below the EAR for calcium (ranging from 10.2% to 12.8% unit decreases, *p* < 0.01 and *p* < 0.05, with each quartile increase in added sugars, depending on age), magnesium (from 8.9% to 12.3% unit decreases, *p* < 0.01 and *p* < 0.05) and vitamin A (from 10.9% to 14.8% unit decreases, *p* < 0.01), with the lowest unit decrease among that 71+ y. Additionally, among those 19–50 y and 71+ y, higher added sugars intake was associated with a higher percentage of individuals with intakes above the AI for potassium (11.8% and 9.2% unit increases, respectively, *p* < 0.05); and among those age 71+ y, there was a lower percentage of individuals with intakes below the EAR for vitamin D (1.7% unit decrease, *p* < 0.01, with every quartile increase in added sugars).

## 4. Discussion

Our analysis of NHANES 2003–2018 cross-sectional data revealed significant associations between added sugars intakes from various beverage sources and indicators of adequacy for several nutrients among US adults. Added sugars intakes from soft drinks were associated with higher percentages below the EAR for calcium, magnesium, and vitamins A, C, and E and lower percentages above the AI for potassium and dietary fiber, with some variation by age group. Associations for calcium, magnesium, and vitamins A and C followed a stepwise pattern, with percentages below the EAR being higher with every change to a higher added sugars quantile of soft drinks. In contrast, added sugars intakes from fruit drinks and flavored milk were associated with lower percentages below the EAR for vitamin C (fruit drinks) and for calcium, magnesium, and vitamin A (flavored milk) and higher percentages above the AI for potassium (flavored milk), with differences also following a stepwise pattern. Mixed associations were observed for sports and energy drinks among adults aged 19–50 y and 51–70 y, with added sugars intakes from these beverages being associated with a higher percentage below the EAR for vitamin A and a lower percentage above the AI for dietary fiber (among those age 19–50 y only), and lower percentages below the EAR for calcium and vitamins B12 and E. For context, looking at the rest of the diet (excluding beverages with added sugars), higher added sugars intake was associated with lower percentages below the EAR for calcium, magnesium, and vitamin A and a higher percentage above the AI for potassium, all in a stepwise fashion across quartiles of added sugars intake.

We are not able to draw direct comparisons of our results to those of others due to the scarcity of studies examining beverages with added sugars and nutrient adequacy among adults. Two studies examined nutrient intakes in Canadian adults or nutrient adequacy in U.S. adults; however, one analysis was on total sugar intake, but not added sugars intake [13], and the second analysis was on added sugars intake as a whole, but not individual beverage sources [14]. Another study of added sugars in older Australians examined nutrient intakes; however, added sugars sources were examined at an aggregate level [15], unlike our analysis of individual sources.

Our study provides a novel contribution to the literature because it reveals associations between added sugars intakes from specific beverage choices and nutrient adequacy, and these associations are generally reflective of the different nutrient compositions of those beverages. For example, soft drinks typically provide calories but not essential nutrients and, thus, may displace other beverage or food choices that provide some of the nutrients we examined. Consumption of soft drinks has been associated with poorer diet quality [16,17], and thus, the differences in indicators of nutrient adequacy we observed (higher percentages below the EAR or lower percentages above the AI) with higher levels of consumption might also be partly due to lower diet quality. On the other hand, fruit drinks provide vitamin C, and flavored milk provides calcium, magnesium, potassium, and vitamins A and D intakes, and these contributions align with our observations on associations with nutrient adequacy. For fruit drinks, 42% to 58% of adults who did not consume fruit drinks had vitamin C intakes below the EAR compared to 2% among adults who were in the highest added sugars quantile of fruit drink consumption. Similarly, among younger adults (19–50 y), 36% of those who did not consume flavored milk had calcium intakes below the EAR compared to 3% of those in the highest quantile of consumption. Furthermore, all associations (both positive and inverse) were in a stepwise manner, suggesting these may be direct associations relating to nutrient displacement and/or contribution; however, some of these associations may be influenced by other components of the diet.

The mixed associations we observed for sports and energy drinks and the lack of any association for coffee and tea likely reflect their variable nutrient compositions and/or their different uses in the diet compared to the other beverage sources we examined. Consumption of sports and energy drinks was more prevalent among younger adults (19–50 y) compared to the other age groups, though still relatively low, at 9% (versus 23% for fruit drinks and 49% for soft drinks), yet differences in indicators of nutrient adequacy were observed between consumers and non-consumers in this younger age group. For example, the percentage with intakes below the EAR for calcium was almost 10% units lower for consumers versus non-consumers of sports and energy drinks, while the percentage with intakes below the EAR for vitamin A was 8% higher. These associations in opposite directions may reflect the variable nutrient composition of beverages within the sports and energy drink category compared to other beverage groups, as well as the degree to which sports and energy drinks may replace other beverages. As for coffee and tea, consumption was similar to fruit drinks in terms of both reported consumption and mean added sugars intake among consumers, yet no associations were observed between added sugars intake from coffee and tea beverages and indicators of nutrient adequacy, suggesting these beverages are not major contributors to nutrient intakes nor are they displacing to any significant degree other beverages with a different nutrient profile. However, because the WWEIA coffee and tea category does not include sugars added by consumers to plain coffee and tea, our analysis was only able to capture added sugars from pre-sweetened, ready-to-drink coffee and tea beverages, and thus significant contributions of plain coffee and tea to potassium intakes, especially among older adults (71+ y) [18] would not have been apparent in our nutrient adequacy results.

The associations we observed between added sugars intakes from the rest of the diet (excluding beverages with added sugars) and nutrient adequacy were consistent with the patterns we observed for fruit drinks and flavored milk. Some of the top sources of added sugars from the rest of the diet would be grain-based and dairy-based groups (i.e., sweet bakery products, breakfast cereals, bars, and frozen desserts) [3], which could directly contribute to nutrient intakes and/or might typically be consumed with a nutrient-dense food or beverage (e.g., cereal and milk). Our findings on the rest of the diet, as well as on fruit drinks and flavored milk, are in line with other research examining sources of added sugars in relation to diet quality and health [19,20], which also highlights the importance of considering the nutritional composition of sources when examining added sugars intakes.

The main strength of our study is the pooling of data from eight cycles of NHANES, which enabled us to examine associations between added sugars intake from a disaggregated group of beverages with varying nutrient compositions. As such, our results make a novel contribution to the literature, overcoming a limitation of examinations of aggregate groupings, which tend to vary in definition and thus limit comparisons across studies. However, due to the cross-sectional nature of NHANES data, we cannot make any conclusions about cause-and-effect relationships between added sugars intake from various beverages and nutrient adequacy. Additionally, because our nutrient adequacy estimates are based on data pooled over a 16-y time span, we cannot infer anything about the prevalence of inadequacy for a particular group at a particular time. Finally, as with all studies using self-reported dietary intake data, ours is limited by both random and systematic errors of reporting. Random errors were adjusted for using 2 d of dietary recall data and the NCI method to estimate UI distributions for all the nutrients we examined. Regarding systematic errors, assuming these errors were evenly distributed across quantiles of added sugars intake (e.g., similar levels of underreporting across quantiles), it is unlikely they would have affected our interpretations of differences in nutrient adequacy across quantiles.

## 5. Conclusions

Our results showed significant associations between added sugars intakes from beverages with added sugars and indicators of adequacy for several nutrients that are under-consumed among adults in the US. The direction of the associations varied by added sugars source, negative for some (i.e., soft drinks) in terms of greater percentages of adults not meeting a defined threshold of nutrient adequacy with higher added sugars intakes, and positive for others (i.e., fruit drinks, flavored milk, rest of the diet) in terms of lower percentages of adults not meeting nutrient thresholds. Taken together, our results suggest that the nutritional composition of the added sugars source, as well as its use in the diet, are important mediators in the relationship between added sugars intake and nutrient adequacy. The contribution of different added sugars sources to nutrient intakes is a critical consideration in developing population-based dietary recommendations, particularly for older adults (≥60 y), who are a nutritionally vulnerable group. Future research might include continued monitoring of changes in added sugars intakes and associations with nutrient adequacy in the context of the total diet and also focusing on the most vulnerable groups, such as older adults.

## Figures and Tables

**Table 1:** Description of added sugars sources using WWEIA food categories.

Added Sugars Source	Description
Sweetened Beverages	soft drinks; fruit drinks; sports and energy drinks; nutritional beverages; smoothies and grain drinks
Soft Drinks	
Fruit Drinks	
Sports and Energy Drinks	
Coffee and Tea	coffee; tea (pre-sweetened and plain)
Flavored Milk	flavored milk: whole; reduced fat; low-fat; nonfat
Rest of the Diet *	milk and dairy (flavored milk excluded); protein foods; mixed dishes; grains; snacks and sweets; fruit; vegetables; beverages (sweetened beverages and coffee and tea excluded); alcoholic beverages; water, fats and oils; condiments and sauces; sugars; baby foods and formulas; other

* All foods and beverages except sweetened beverages (soft drinks; fruit drinks; sports and energy drinks; nutritional beverages; smoothies and grain drinks), coffee and tea, and flavored milk. WWEIA, What We Eat in America [9].

**Table 2:** Percent of the population reporting intake (consumers) of beverage sources of added sugars and from the rest of the diet among adults from the pooled sample (NHANES 2003–2018, n = 35,128).

Added Sugars Source	Consumers (%) Mean ± SE		
	19–50 y (n = 18,110)	51–70 y (n = 11,379)	71+ y (n = 5639)
Sweetened Beverages	63.68 ± 0.73	46.06 ± 0.92	39.49 ± 0.87
Soft Drinks	48.83 ± 0.78	31.86 ± 0.90	21.98 ± 0.78
Fruit Drinks	23.16 ± 0.61	16.64 ± 0.55	16.55 ± 0.67
Sports and Energy Drinks	9.33 ± 0.41	3.47 ± 0.27	1.95 ± 0.26
Coffee and Tea	21.43 ± 0.60	16.74 ± 0.76	13.32 ± 0.80
Flavored Milk	4.85 ± 0.24	3.35 ± 0.26	4.35 ± 0.36
Rest of the Diet	99.48 ± 0.08	99.81 ± 0.05	99.85 ± 0.06

**Table 3:** Added sugars intake (% kcal) from beverage sources and the rest of the diet among adults from the pooled sample (NHANES 2003–2018, n = 35,128).

	Added Sugars (% kcal) Mean ± SE					
	19–50 y (n = 18,110)		51–70 y (n = 11,379)		71+ y (n = 5639)	
	All Individuals	Consumers Only	All Individuals	Consumers Only	All Individuals	Consumers Only
Sweetened Beverages	6.19 ± 0.14	9.73 ± 0.16	3.42 ± 0.11	7.43 ± 0.17	2.22 ± 0.07	5.63 ± 0.12
Soft Drinks	4.52 ± 0.13	9.26 ± 0.17	2.45 ± 0.10	7.70 ± 0.21	1.38 ± 0.06	6.29 ± 0.17
Fruit Drinks	1.15 ± 0.04	4.95 ± 0.10	0.75 ± 0.04	4.49 ± 0.15	0.66 ± 0.04	3.98 ± 0.17
Sports and Energy Drinks	0.45 ± 0.02	4.86 ± 0.18	0.14 ± 0.01	4.16 ± 0.32	0.06 ± 0.01	3.06 ± 0.43
Coffee and Tea	1.07 ± 0.04	5.00 ± 0.14	0.93 ± 0.07	5.57 ± 0.29	0.68 ± 0.08	5.12 ± 0.43
Flavored Milk	0.12 ± 0.01	2.40 ± 0.10	0.08 ± 0.01	2.35 ± 0.13	0.11 ± 0.01	2.54 ± 0.10
Rest of the Diet *	6.92 ± 0.06	6.95 ± 0.06	7.91 ± 0.07	7.92 ± 0.07	8.82 ± 0.09	8.84 ± 0.09
Total Added Sugars	14.3 ± 0.16		12.3 ± 0.15		11.8 ± 0.16	

* Given almost the entire population consumes added sugars from the rest of the diet, mean added sugars values for All Individuals and Consumers Only are almost identical within each age group.

**Table 4:** Quantiles of added sugars intake (% kcal) by beverage source among adults from the pooled sample (NHANES 2003–2018, n = 35,128).

Added Sugars Source	Quantile	19–50 y (n = 18,110) Range (% kcal)	51–70 y (n = 11,379) Range (% kcal)	71+ y (n = 5639) Range (% kcal)
Sweetened Beverages	1 *	0	0	0
	2	>0 to ≤4.90	>0 to ≤3.66	>0 to ≤2.83
	3	>4.90 to ≤10.87	>3.66 to ≤8.31	>2.83 to ≤6.05
	4	>10.87	>8.31	>6.05
Soft Drinks	1 *	0	0	0
	2	>0 to ≤4.76	>0 to ≤3.94	>0 to ≤3.40
	3	>4.76 to ≤10.04	>3.94 to ≤8.35	>3.40 to ≤6.82
	4	>10.04	>8.35	>6.82
Fruit Drinks	1 *	0	0	0
	2	>0 to ≤2.39	>0 to ≤2.22	>0 to ≤1.89
	3	>2.39 to ≤5.15	>2.22 to ≤4.90	>1.89 to ≤4.19
	4	>5.15	>4.90	>4.19
Sports and Energy Drinks	1 *	0	0	0
	2	>0 to ≤2.46	>0 to ≤2.34	>0 to ≤1.28
	3	>2.46 to ≤4.90	>2.34 to ≤4.25	>1.28 to ≤3.31
	4	>4.90	>4.25	>3.31
Coffee and Tea	1 *	0	0	0
	2	>0 to ≤2.36	>0 to ≤2.40	>0 to ≤2.26
	3	>2.36 to ≤5.14	>2.40 to ≤5.27	>2.26 to ≤5.05
	4	>5.14	>5.27	>5.05
Flavored Milk	1 *	0	0	0
	2	>0 to ≤1.37	>0 to ≤1.41	>0 to ≤1.75
	3	>1.37 to ≤2.39	>1.41 to ≤2.42	>1.75 to ≤2.81
	4	>2.39	>2.42	>2.81

* Quantile 1 represents non-consumers; the remaining sample of those reporting intake (consumers) was divided into tertiles (quantiles 2, 3, and 4).

**Table 5:** Trends in % <EAR (>AI ^1^) for selected nutrients by beverage source of added sugars among adults (19–50 y, n = 18,110) from the pooled sample (NHANES 2003–2018, n = 35,128); weighted n’s are shown in the table.

Sweetened Beverages							
	Q1 ^2^ n = 45,870,329 % <EAR (SE)	Q2 n = 26,738,412 % <EAR (SE)	Q3 n = 26,858,208 % <EAR (SE)	Q4 n = 26,819,883 % <EAR (SE)	Quantile Trend ^3^ Beta (SE), *p*	Q1 vs. Q2,3,4 ^4^ Beta (SE), *p*	Q2–Q4 Trend ^5^ Beta (SE), *p*
Calcium	33.82 (1.08)	34.32 (1.45)	34.01 (1.58)	35.60 (1.49)	0.49 (0.24), 0.18	0.83 (0.88), 0.44	0.65 (0.55), 0.45
Magnesium	44.86 (1.15)	51.31 (1.49)	58.22 (1.54)	65.32 (1.34)	6.81 (0.10), 0.00 ^†^	13.56 (7.19), 0.20	7.00 (0.06), 0.01 ^†^
Potassium ^1^	35.54 (1.14)	30.94 (1.39)	28.07 (1.45)	24.66 (1.32)	−3.59 (0.23), 0.00 ^†^	−7.71 (3.23), 0.14	−3.14 (0.16), 0.03 ^§^
Vitamin A	40.88 (1.44)	48.10 (1.72)	56.22 (1.61)	62.29 (1.64)	7.25 (0.26), 0.00 ^†^	14.82 (7.30), 0.18	7.09 (0.60), 0.05
Vitamin B12	5.51 (0.55)	3.69 (0.81)	2.96 (0.66)	2.45 (0.55)	−1.02 (0.21), 0.04 ^§^	−2.49 (0.64), 0.06	−0.62 (0.07), 0.07
Vitamin C	49.15 (1.42)	43.07 (1.70)	47.48 (1.72)	51.36 (1.61)	0.84 (1.68), 0.67	−1.76 (4.25), 0.72	4.14 (0.16), 0.02 ^§^
Vitamin D	95.62 (0.54)	95.24 (0.67)	96.37 (0.69)	96.85 (0.49)	0.45 (0.19), 0.15	0.55 (0.84), 0.58	0.80 (0.19), 0.15
Vitamin E	81.91 (0.97)	80.86 (1.65)	88.96 (1.24)	89.78 (1.12)	3.06 (1.12), 0.11	4.75 (5.04), 0.45	4.42 (2.12), 0.28
Dietary Fiber ^1^	5.09 (0.48)	2.90 (0.45)	1.46 (0.29)	0.71 (0.16)	−1.49 (0.22), 0.02 ^§^	−3.43 (1.14), 0.10	−1.09 (0.20), 0.12
Protein	1.45 (0.21)	1.17 (0.38)	1.28 (0.32)	0.84 (0.23)	−0.17 (0.07), 0.12	−0.35 (0.24), 0.28	−0.17 (0.16), 0.48
**Soft Drinks**							
	**Q1 ^2^** **n = 64,624,982** **% <EAR (SE)**	**Q2** **n = 20,527,842** **% <EAR (SE)**	**Q3** **n = 20,569,006** **% <EAR (SE)**	**Q4** **n = 20,565,002** **% <EAR (SE)**	**Quantile Trend ^3^** **Beta (SE), *p***	**Q1 vs. Q2,3,4 ^4^** **Beta (SE), *p***	**Q2–Q4 Trend ^5^** **Beta (SE), *p***
Calcium	32.55 (0.93)	36.62 (1.67)	34.77 (1.70)	36.86 (1.70)	1.34 (0.67), 0.19	3.53 (0.96), 0.07	0.09 (1.14), 0.95
Magnesium	44.52 (1.04)	57.34 (1.58)	62.67 (1.76)	70.27 (1.53)	8.68 (0.98), 0.01 ^§^	18.73 (5.44), 0.07	6.45 (0.65), 0.06
Potassium ^1^	36.21 (0.96)	26.49 (1.55)	25.38 (1.53)	21.56 (1.42)	−4.99 (1.12), 0.05 ^§^	−11.66 (2.16), 0.03 ^§^	−2.45 (0.78), 0.20
Vitamin A	40.69 (1.27)	56.72 (1.80)	59.54 (2.07)	65.52 (2.14)	8.53 (1.80), 0.04 ^§^	19.79 (3.75), 0.03 ^§^	4.38 (0.91), 0.13
Vitamin B12	4.92 (0.51)	2.74 (0.59)	3.26 (0.73)	2.04 (0.60)	−0.92 (0.31), 0.10	−2.23 (0.51), 0.05 ^§^	−0.34 (0.50), 0.62
Vitamin C	41.17 (1.21)	47.71 (1.71)	53.32 (2.01)	66.69 (1.89)	7.89 (1.05), 0.02 ^§^	14.48 (8.14), 0.22	9.44 (2.24), 0.15
Vitamin D	95.46 (0.44)	95.90 (0.87)	97.09 (0.57)	96.61 (0.56)	0.49 (0.18), 0.12	1.06 (0.51), 0.17	0.37 (0.48), 0.59
Vitamin E	79.72 (0.95)	88.28 (1.59)	92.54 (1.21)	91.65 (1.31)	4.57 (1.33), 0.08	11.06 (1.91), 0.03 ^§^	1.72 (1.49), 0.46
Dietary Fiber ^1^	4.90 (0.42)	1.45 (0.33)	0.94 (0.23)	0.33 (0.09)	−1.63 (0.46), 0.07	−3.98 (0.47), 0.01 ^§^	−0.56 (0.03), 0.03 ^§^
Protein	1.43 (0.20)	1.04 (0.30)	1.09 (0.31)	0.76 (0.28)	−0.21 (0.05), 0.06	−0.46 (0.15), 0.09	−0.14 (0.11), 0.44
**Fruit Drinks**							
	**Q1 ^2^** **n = 97,041,663** **% <EAR (SE)**	**Q2** **n = 9,649,034** **% <EAR (SE)**	**Q3** **n = 9,843,621** **% <EAR (SE)**	**Q4** **n = 9,752,514** **% <EAR (SE)**	**Quantile Trend ^3^** **Beta (SE), *p***	**Q1 vs. Q2,3,4 ^4^** **Beta (SE), *p***	**Q2–Q4 Trend ^5^** **Beta (SE), *p***
Calcium	34.26 (0.85)	36.09 (2.47)	36.67 (2.01)	30.23 (2.03)	−0.60 (1.00), 0.61	−0.15 (2.48), 0.96	−3.05 (2.02), 0.37
Magnesium	53.27 (0.98)	53.35 (2.84)	57.45 (2.18)	48.36 (2.05)	−0.65 (1.32), 0.67	−0.42 (3.17), 0.91	−2.73 (3.79), 0.60
Potassium ^1^	30.44 (0.83)	29.20 (2.44)	27.00 (2.06)	38.35 (2.04)	1.42 (1.61), 0.47	1.43 (4.21), 0.77	4.82 (3.90), 0.43
Vitamin A	49.66 (1.08)	46.04 (3.52)	54.59 (2.58)	52.08 (2.18)	1.07 (1.11), 0.43	1.44 (2.89), 0.67	2.83 (3.18), 0.54
Vitamin B12	4.10 (0.33)	2.74 (1.05)	6.38 (1.29)	2.51 (1.17)	−0.14 (0.64), 0.85	−0.24 (1.47), 0.88	−0.25 (2.16), 0.93
Vitamin C	57.84 (1.06)	26.50 (3.38)	10.71 (3.91)	1.66 (1.05)	−20.36 (2.70), 0.02 ^§^	−45.76 (8.45), 0.03 ^§^	−12.30 (1.94), 0.10
Vitamin D	95.91 (0.36)	96.35 (0.90)	95.68 (1.00)	96.89 (0.90)	0.22 (0.16), 0.31	0.42 (0.42), 0.42	0.30 (0.54), 0.68
Vitamin E	84.39 (0.68)	82.88 (2.31)	87.38 (2.60)	86.53 (1.65)	0.83 (0.54), 0.27	1.33 (1.57), 0.49	1.73 (1.54), 0.46
Dietary Fiber ^1^	3.00 (0.28)	3.05 (0.73)	1.27 (0.36)	2.62 (0.65)	−0.30 (0.27), 0.38	−0.70 (0.62), 0.38	−0.16 (0.90), 0.89
Protein	1.27 (0.16)	0.98 (0.45)	1.50 (0.52)	0.78 (0.42)	−0.10 (0.11), 0.45	−0.19 (0.25), 0.53	−0.12 (0.36), 0.79
**Sports and Energy Drinks**							
	**Q1 ^2^** **n = 114,501,042** **% <EAR (SE)**	**Q2** **n = 3,911,941** **% <EAR (SE)**	**Q3** **n = 3,884,247** **% <EAR (SE)**	**Q4** **n = 3,989,602** **% <EAR (SE)**	**Quantile Trend ^3^** **Beta (SE), *p***	**Q1 vs. Q2,3,4 ^4^** **Beta (SE), *p***	**Q2–Q4 Trend ^5^** **Beta (SE), *p***
Calcium	35.08 (0.77)	24.83 (4.20)	24.94 (2.74)	26.60 (3.69)	−3.99 (1.48), 0.12	−9.65 (0.59), 0.00 ^†^	0.87 (0.45), 0.30
Magnesium	53.39 (0.90)	51.62 (4.27)	52.95 (4.48)	51.83 (3.83)	−0.52 (0.28), 0.21	−1.25 (0.44), 0.10	0.12 (0.72), 0.89
Potassium ^1^	30.81 (0.72)	25.37 (5.39)	32.39 (3.76)	33.33 (4.30)	0.41 (1.21), 0.77	−0.51 (2.64), 0.86	4.02 (1.78), 0.27
Vitamin A	49.35 (0.99)	56.08 (5.43)	60.29 (4.40)	55.45 (4.34)	3.37 (1.33), 0.13	7.97 (1.60), 0.04 ^§^	−0.26 (2.65), 0.94
Vitamin B12	4.66 (0.33)	0.02 (0.17)	0.45 (0.54)	0.00 (0.02)	−1.92 (0.59), 0.08	−4.50 (0.15), 0.00 ^†^	−0.01 (0.25), 0.98
Vitamin C	47.94 (1.01)	48.38 (5.37)	53.97 (4.68)	55.32 (3.66)	2.49 (0.46), 0.03 ^§^	4.55 (2.23), 0.18	3.50 (1.24), 0.22
Vitamin D	95.99 (0.31)	96.14 (1.14)	96.25 (1.44)	95.09 (2.64)	−0.14 (0.15), 0.45	−0.15 (0.38), 0.74	−0.52 (0.37), 0.40
Vitamin E	85.24 (0.63)	79.40 (5.64)	83.77 (4.37)	73.56 (4.60)	−3.08 (1.20), 0.12	−6.18 (3.08), 0.18	−2.82 (4.27), 0.63
Dietary Fiber ^1^	3.06 (0.25)	0.92 (0.75)	1.00 (0.52)	1.28 (0.76)	−0.82 (0.31), 0.11	−2.00 (0.11), 0.00 ^†^	0.18 (0.05), 0.19
Protein	1.21 (0.14)	0.68 (0.92)	1.71 (1.15)	2.50 (0.90)	0.31 (0.18), 0.23	0.40 (0.55), 0.54	0.91 (0.07), 0.05
**Coffee and Tea**							
	**Q1 ^2^** **n = 99,219,698** **% <EAR (SE)**	**Q2** **n = 8,638,066** **% <EAR (SE)**	**Q3** **n = 9,403,285** **% <EAR (SE)**	**Q4** **n = 9,025,782** **% <EAR (SE)**	**Quantile Trend ^3^** **Beta (SE), *p***	**Q1 vs. Q2,3,4 ^4^** **Beta (SE), *p***	**Q2–Q4 Trend ^5^** **Beta (SE), *p***
Calcium	34.03 (0.87)	36.70 (2.36)	34.64 (2.56)	35.84 (3.13)	0.62 (0.44), 0.30	1.67 (0.68), 0.13	−0.43 (0.96), 0.73
Magnesium	52.98 (0.94)	54.61 (2.37)	55.95 (2.74)	53.62 (2.86)	0.65 (0.49), 0.31	1.78 (0.77), 0.15	−0.49 (1.08), 0.73
Potassium ^1^	30.48 (0.85)	30.57 (2.55)	28.22 (2.59)	37.20 (2.54)	1.15 (1.20), 0.44	1.39 (3.02), 0.69	3.29 (3.36), 0.51
Vitamin A	49.43 (1.02)	50.86 (2.80)	51.60 (2.81)	54.08 (3.17)	1.41 (0.16), 0.01 ^§^	2.73 (1.08), 0.13	1.60 (0.51), 0.20
Vitamin B12	4.23 (0.31)	4.38 (1.26)	3.92 (1.32)	1.44 (0.65)	−0.65 (0.32), 0.18	−0.95 (1.02), 0.45	−1.47 (0.60), 0.25
Vitamin C	46.93 (1.03)	43.97 (3.48)	55.08 (2.48)	60.68 (3.00)	4.06 (1.47), 0.11	6.30 (5.48), 0.37	8.36 (1.63), 0.12
Vitamin D	95.74 (0.37)	96.56 (1.00)	96.93 (0.77)	97.34 (0.64)	0.56 (0.06), 0.01 ^†^	1.20 (0.25), 0.04 ^§^	0.39 (0.01), 0.02 ^§^
Vitamin E	84.33 (0.78)	85.19 (2.62)	86.28 (2.36)	87.28 (1.81)	0.98 (0.02), 0.00 ^†^	1.92 (0.68), 0.11	1.05 (0.03), 0.02 ^§^
Dietary Fiber ^1^	3.19 (0.29)	2.95 (0.75)	2.06 (0.81)	0.66 (0.36)	−0.73 (0.14), 0.04 ^§^	−1.28 (0.74), 0.23	−1.15 (0.15), 0.08
Protein	1.21 (0.15)	1.79 (0.65)	0.99 (0.53)	0.85 (0.32)	−0.08 (0.14), 0.63	0.00 (0.33), 0.99	−0.47 (0.20), 0.25
**Flavored Milk**							
	Q1 ^2^ n = 120,167,033 % <EAR (SE)	**Q2** **n = 2,033,860** **% <EAR (SE)**	**Q3** **n = 2,024,315** **% <EAR (SE)**	**Q4** **n = 2,061,624** **% <EAR (SE)**	**Quantile Trend ^3^** **Beta (SE), *p***	**Q1 vs. Q2,3,4 ^4^** **Beta (SE), *p***	**Q2–Q4 Trend ^5^** **Beta (SE), *p***
Calcium	35.62 (0.78)	22.08 (4.14)	7.64 (3.11)	3.40 (2.18)	−11.85 (1.10), 0.01 ^†^	−24.51 (5.82), 0.05	−9.36 (2.94), 0.19
Magnesium	54.10 (0.87)	43.66 (5.38)	33.50 (8.79)	25.54 (5.31)	−9.81 (0.28), 0.00 ^†^	−19.81 (5.39), 0.07	−9.07 (0.64), 0.04 ^§^
Potassium ^1^	29.86 (0.72)	38.17 (6.92)	49.06 (6.61)	63.15 (5.19)	10.48 (0.65), 0.00 ^†^	20.19 (7.43), 0.11	12.48 (0.92), 0.05 ^§^
Vitamin A	51.08 (0.99)	39.34 (5.53)	31.50 (5.99)	19.03 (3.68)	−10.50 (0.38), 0.00 ^†^	−21.06 (6.07), 0.07	−10.15 (1.33), 0.08
Vitamin B12	4.32 (0.30)	1.50 (1.18)	0.25 (0.42)	0.02 (0.06)	−1.70 (0.30), 0.03 ^§^	−3.72 (0.47), 0.02 ^§^	−0.74 (0.29), 0.24
Vitamin C	48.45 (0.97)	51.21 (4.99)	40.81 (8.50)	49.42 (5.25)	−0.68 (1.50), 0.69	−1.28 (3.29), 0.74	−0.93 (5.47), 0.89
Vitamin D	96.52 (0.29)	93.18 (2.51)	94.16 (5.65)	64.50 (7.78)	−7.48 (3.16), 0.14	−12.51 (9.96), 0.34	−14.29 (8.82), 0.35
Vitamin E	84.66 (0.65)	78.89 (4.83)	89.97 (4.26)	84.83 (3.60)	0.39 (1.50), 0.82	−0.14 (3.29), 0.97	3.00 (4.67), 0.64
Dietary Fiber ^1^	2.84 (0.24)	2.70 (1.73)	1.64 (1.20)	4.47 (2.21)	0.17 (0.37), 0.69	0.10 (0.84), 0.92	0.88 (1.12), 0.58
Protein	1.29 (0.16)	0.45 (0.52)	0.16 (0.26)	0.08 (0.10)	−0.48 (0.09), 0.03 ^§^	−1.06 (0.11), 0.01 ^§^	−0.18 (0.06), 0.20

EAR, estimated average requirement; AI, adequate intake. ^1^ >AI for potassium and dietary fiber. ^2^ Quantile 1 represents non-consumers; the remaining sample of those reporting intake (consumers) was divided into tertiles (quantiles 2, 3, and 4). ^3^ From regression analysis among all individuals, test for trend. ^4^ From regression analysis among all individuals, test for differences between non-consumers (Q1) and consumers (Q2,3,4). ^5^ From regression analysis among consumers (Q2–Q4), test for trend. ^†^ Statistically significant at *p* < 0.01; ^§^ Statistically significant at *p* < 0.05.

**Table 6:** Trends in % <EAR (>AI ^1^) for selected nutrients by beverage source of added sugars among adults (51–70 y, n = 11,379) from the pooled sample (NHANES 2003–2018, n = 35,128); weighted n’s are shown in the table.

Sweetened Beverages							
	Q1 ^2^ n = 37,742,394 % <EAR (SE)	Q2 n = 9,641,654 % <EAR (SE)	Q3 n = 10,517,451 % <EAR (SE)	Q4 n = 12,072,235 % <EAR (SE)	Quantile Trend ^3^ Beta (SE), *p*	Q1 vs. Q2,3,4 ^4^ Beta (SE), *p*	Q2–4 Trend ^5^ Beta (SE), *p*
Calcium	54.98 (1.14)	49.43 (2.17)	51.25 (2.21)	56.20 (2.12)	−0.07 (1.42), 0.97	−2.42 (2.93), 0.49	3.45 (0.90), 0.16
Magnesium	49.30 (1.33)	51.85 (2.08)	55.95 (2.11)	68.36 (1.67)	5.69 (1.36), 0.05	10.06 (7.20), 0.30	8.42 (2.41), 0.18
Potassium ^1^	40.13 (1.27)	38.26 (2.04)	35.83 (1.82)	27.45 (1.83)	−3.77 (0.91), 0.05	−6.70 (4.75), 0.29	−5.52 (1.72), 0.19
Vitamin A	39.50 (1.49)	39.38 (2.10)	46.15 (2.16)	53.12 (2.41)	4.30 (1.02), 0.052	7.25 (5.65), 0.33	6.87 (0.06), 0.01 ^†^
Vitamin B12	5.23 (0.55)	3.92 (0.90)	3.96 (0.87)	3.39 (0.82)	−0.62 (0.15), 0.05	−1.50 (0.27), 0.03 ^§^	−0.28 (0.18), 0.37
Vitamin C	45.12 (1.26)	35.45 (2.17)	39.46 (2.39)	45.82 (2.10)	−0.40 (2.24), 0.88	−4.48 (4.34), 0.41	5.23 (0.68), 0.08
Vitamin D	95.82 (0.50)	93.40 (0.92)	95.02 (1.01)	96.14 (0.64)	0.01 (0.54), 0.99	−0.86 (1.12), 0.53	1.36 (0.15), 0.07
Vitamin E	82.49 (1.05)	82.53 (2.00)	85.51 (1.75)	91.29 (1.22)	2.63 (0.77), 0.08	4.29 (3.71), 0.37	4.44 (0.81), 0.11
Dietary Fiber ^1^	15.99 (1.01)	13.91 (1.47)	9.12 (1.17)	4.47 (0.68)	−3.76 (0.38), 0.01 ^§^	−7.18 (3.88), 0.21	−4.72 (0.04), 0.01 ^†^
Protein	1.98 (0.27)	2.02 (0.56)	1.71 (0.39)	1.55 (0.39)	−0.14 (0.04), 0.06	−0.23 (0.19), 0.34	−0.23 (0.04), 0.11
**Soft Drinks**							
	Q1 ^2^ n = 47,679,443 % <EAR (SE)	**Q2** **n = 6,587,851** **% <EAR (SE)**	**Q3** **n = 7,594,210** **% <EAR (SE)**	**Q4** **n = 8,112,229** **% <EAR (SE)**	**Quantile Trend ^3^** **Beta (SE), *p***	**Q1 vs. Q2,3,4 ^4^** **Beta (SE), *p***	**Q2–4 Trend ^5^** **Beta (SE), *p***
Calcium	52.72 (0.95)	51.97 (2.43)	53.93 (2.50)	58.97 (2.54)	1.60 (0.73), 0.16	2.28 (2.58), 0.47	3.52 (0.91), 0.16
Magnesium	47.87 (1.18)	59.62 (2.57)	63.70 (2.81)	76.48 (1.72)	9.15 (0.84), 0.01 ^†^	18.85 (6.30), 0.10	8.49 (2.58), 0.19
Potassium ^1^	41.76 (1.10)	30.86 (2.06)	28.40 (2.21)	21.42 (2.00)	−6.86 (0.85), 0.02 ^§^	−14.93 (3.50), 0.05	−4.75 (1.34), 0.17
Vitamin A	38.20 (1.20)	49.85 (2.56)	48.86 (3.09)	58.27 (3.02)	6.45 (1.23), 0.03 ^§^	14.13 (3.74), 0.06	4.27 (3.08), 0.40
Vitamin B12	5.03 (0.53)	2.95 (0.72)	4.59 (1.10)	3.19 (1.03)	−0.55 (0.36), 0.27	−1.43 (0.64), 0.16	0.10 (0.90), 0.93
Vitamin C	38.81 (1.06)	46.49 (2.54)	49.10 (3.18)	60.60 (2.76)	6.70 (0.84), 0.02 ^§^	13.34 (5.38), 0.13	7.11 (2.63), 0.23
Vitamin D	95.02 (0.44)	94.90 (1.29)	96.56 (0.76)	96.29 (0.73)	0.51 (0.19), 0.12	0.93 (0.63), 0.28	0.68 (0.57), 0.44
Vitamin E	81.17 (0.96)	87.26 (2.14)	92.06 (1.38)	93.40 (1.28)	4.51 (0.61), 0.02 ^§^	9.85 (2.28), 0.05 ^§^	3.05 (1.03), 0.21
Dietary Fiber ^1^	16.23 (0.92)	8.21 (1.19)	6.58 (1.17)	2.22 (0.43)	−4.80 (0.68), 0.02 ^§^	−10.60 (2.21), 0.04 ^§^	−3.01 (0.81), 0.17
Protein	1.93 (0.24)	1.69 (0.50)	1.89 (0.45)	1.94 (0.57)	−0.01 (0.05), 0.89	−0.08 (0.09), 0.48	0.12 (0.05), 0.23
**Fruit Drinks**							
	**Q1 ^2^** **n = 58,332,159** **% <EAR (SE)**	**Q2** **n = 3,528,538** **% <EAR (SE)**	**Q3** **n = 3,672,186** **% <EAR (SE)**	**Q4** **n = 4,440,850** **% <EAR (SE)**	**Quantile Trend ^3^** **Beta (SE), *p***	**Q1 vs. Q2,3,4 ^4^** **Beta (SE), *p***	**Q2–4 Trend ^5^** **Beta (SE), *p***
Calcium	53.79 (0.90)	52.06 (3.03)	57.72 (3.21)	47.84 (2.74)	−0.96 (1.35), 0.55	−1.42 (3.24), 0.70	−2.30 (4.43), 0.70
Magnesium	53.82 (1.10)	52.78 (2.76)	55.87 (3.45)	47.75 (2.57)	−1.20 (1.03), 0.37	−1.86 (2.69), 0.56	−2.65 (3.20), 0.56
Potassium ^1^	36.53 (1.03)	36.48 (2.52)	34.58 (2.94)	46.80 (2.63)	2.14 (1.54), 0.30	3.08 (4.33), 0.55	5.33 (4.03), 0.41
Vitamin A	43.02 (1.21)	36.13 (3.33)	42.69 (3.89)	42.40 (3.38)	−0.50 (1.26), 0.73	−2.49 (2.35), 0.40	3.05 (1.95), 0.36
Vitamin B12	4.48 (0.42)	3.06 (1.48)	8.13 (2.22)	2.31 (1.10)	−0.10 (0.90), 0.92	−0.04 (2.04), 0.99	−0.50 (3.10), 0.90
Vitamin C	49.89 (1.14)	17.55 (2.81)	3.79 (2.58)	2.32 (1.54)	−18.45 (3.62), 0.04 ^§^	−42.33 (5.35), 0.02 ^§^	−7.47 (3.51), 0.28
Vitamin D	95.52 (0.34)	92.55 (1.65)	95.42 (1.12)	96.12 (1.47)	−0.01 (0.59), 0.99	−0.75 (1.21), 0.60	1.76 (0.62), 0.21
Vitamin E	83.99 (0.92)	84.15 (2.22)	89.25 (2.16)	84.38 (2.17)	0.77 (0.86), 0.46	1.90 (1.84), 0.41	0.00 (2.84), 1.00
Dietary Fiber ^1^	12.95 (0.77)	11.77 (1.79)	8.31 (1.94)	13.90 (1.82)	−0.43 (0.90), 0.68	−1.53 (1.84), 0.49	1.18 (2.58), 0.73
Protein	1.90 (0.21)	1.99 (0.83)	1.76 (0.73)	1.06 (0.51)	−0.21 (0.09), 0.15	−0.32 (0.31), 0.42	−0.47 (0.13), 0.18
**Sports and Energy Drinks**							
	Q1 ^2^ n = 67,548,696 % <EAR (SE)	**Q2** **n = 831,108** **% <EAR (SE)**	**Q3** **n = 816,301** **% <EAR (SE)**	**Q4** **n = 881,517** **% <EAR (SE)**	**Quantile Trend ^3^** **Beta (SE), *p***	**Q1 vs. Q2,3,4 ^4^** **Beta (SE), *p***	**Q2–4 Trend ^5^** **Beta (SE), *p***
Calcium	54.22 (0.83)	44.33 (9.35)	33.43 (8.40)	33.98 (5.40)	−7.86 (1.19), 0.02 ^§^	−17.43 (3.46), 0.04 ^§^	−4.91 (3.29), 0.38
Magnesium	53.77 (1.03)	63.96 (7.04)	45.65 (8.22)	48.02 (7.25)	−1.81 (2.22), 0.50	−1.93 (5.60), 0.76	−7.48 (5.94), 0.43
Potassium ^1^	36.88 (0.91)	24.95 (8.37)	46.98 (8.14)	42.74 (6.71)	2.00 (2.64), 0.53	2.12 (6.57), 0.78	8.28 (7.54), 0.47
Vitamin A	42.53 (1.16)	53.83 (7.54)	43.40 (8.99)	56.88 (8.69)	4.02 (1.87), 0.16	9.05 (4.21), 0.16	2.09 (6.87), 0.81
Vitamin B12	4.71 (0.41)	0.10 (0.36)	0.33 (0.50)	0.00 (0.07)	−1.90 (0.52), 0.07	−4.57 (0.10), 0.00 ^†^	−0.06 (0.16), 0.77
Vitamin C	43.05 (1.04)	53.01 (8.99)	39.76 (9.13)	49.68 (8.69)	1.64 (1.90), 0.48	4.34 (3.96), 0.39	−1.12 (6.65), 0.89
Vitamin D	95.47 (0.35)	96.58 (1.84)	90.72 (5.91)	90.50 (4.30)	−1.69 (0.54), 0.09	−3.14 (1.95), 0.25	−2.91 (1.62), 0.32
Vitamin E	84.69 (0.83)	78.04 (7.61)	78.11 (8.97)	79.05 (5.01)	−2.50 (0.85), 0.10	−6.24 (0.34), 0.00 ^†^	0.52 (0.25), 0.28
Dietary Fiber ^1^	12.68 (0.64)	7.17 (3.85)	13.69 (6.15)	6.72 (2.47)	−1.54 (1.05), 0.28	−3.55 (2.29), 0.26	−0.54 (3.87), 0.91
Protein	1.87 (0.20)	0.20 (0.64)	1.16 (0.98)	1.98 (1.14)	−0.16 (0.29), 0.64	−0.68 (0.52), 0.32	0.88 (0.04), 0.03 ^§^
**Coffee and Tea**							
	**Q1 ^2^** **n = 58,259,558** **% <EAR (SE)**	**Q2** **n = 3,642,763** **% <EAR (SE)**	**Q3** **n = 3,965,805** **% <EAR (SE)**	**Q4** **n = 4,105,608** **% <EAR (SE)**	**Quantile Trend ^3^** **Beta (SE), *p***	**Q1 vs. Q2,3,4 ^4^** **Beta (SE), *p***	**Q2–4 Trend ^5^** **Beta (SE), *p***
Calcium	52.93 (0.90)	63.97 (2.91)	51.04 (3.68)	57.15 (3.53)	1.37 (2.36), 0.62	4.79 (4.22), 0.37	−3.78 (5.46), 0.61
Magnesium	53.13 (1.06)	59.40 (3.11)	52.39 (3.56)	55.50 (3.38)	0.82 (1.31), 0.60	2.82 (2.29), 0.34	−2.15 (2.91), 0.59
Potassium ^1^	37.16 (0.99)	29.31 (2.72)	38.10 (4.11)	41.15 (3.19)	0.46 (1.91), 0.83	−1.47 (3.97), 0.75	6.04 (1.65), 0.17
Vitamin A	42.00 (1.15)	44.86 (3.75)	44.40 (3.85)	51.03 (4.46)	2.47 (0.63), 0.06	4.54 (2.27), 0.18	2.95 (2.03), 0.38
Vitamin B12	4.37 (0.43)	6.43 (1.78)	3.80 (1.27)	2.83 (1.06)	−0.27 (0.54), 0.66	0.13 (1.20), 0.92	−1.84 (0.48), 0.16
Vitamin C	41.96 (1.06)	37.60 (3.96)	49.06 (3.99)	57.63 (4.53)	4.08 (2.00), 0.18	5.32 (6.42), 0.49	10.07 (0.83), 0.05
Vitamin D	95.09 (0.38)	97.24 (0.89)	95.64 (1.33)	97.19 (0.81)	0.67 (0.37), 0.21	1.61 (0.58), 0.11	−0.09 (0.90), 0.94
Vitamin E	84.00 (0.93)	89.05 (2.41)	84.48 (2.53)	85.45 (2.94)	0.72 (0.99), 0.54	2.48 (1.58), 0.26	−1.91 (1.59), 0.44
Dietary Fiber ^1^	13.18 (0.71)	12.26 (2.16)	11.21 (2.41)	5.79 (1.67)	−1.92 (0.55), 0.07	−3.18 (2.15), 0.28	−3.15 (1.25), 0.24
Protein	1.79 (0.22)	4.47 (1.20)	0.95 (0.43)	1.80 (0.61)	0.06 (0.62), 0.93	0.74 (1.20), 0.60	−1.42 (1.25), 0.46
**Flavored Milk**							
	**Q1 ^2^** **n = 67,632,660** **% <EAR (SE)**	**Q2** **n = 741,030** **% <EAR (SE)**	**Q3** **n = 761,003** **% <EAR (SE)**	**Q4** **n = 839,040** **% <EAR (SE)**	**Quantile Trend ^3^** **Beta (SE), *p***	**Q1 vs. Q2,3,4 ^4^** **Beta (SE), *p***	**Q2–4 Trend ^5^** **Beta (SE), *p***
Calcium	54.48 (0.88)	50.32 (7.71)	26.34 (8.98)	21.19 (6.63)	−11.41 (1.71), 0.02 ^§^	−21.85 (9.22), 0.14	−14.51 (5.27), 0.22
Magnesium	53.87 (1.03)	59.56 (6.99)	43.66 (12.76)	32.13 (6.60)	−5.80 (2.37), 0.13	−8.88 (8.21), 0.39	−13.70 (1.22), 0.06
Potassium ^1^	36.76 (0.92)	36.15 (10.67)	53.99 (9.42)	60.33 (6.48)	7.48 (1.62), 0.04 ^§^	13.42 (7.45), 0.21	12.06 (3.22), 0.17
Vitamin A	43.09 (1.10)	53.03 (7.87)	25.60 (10.51)	20.56 (5.34)	−6.64 (3.31), 0.18	−9.99 (10.35), 0.44	−16.17 (6.27), 0.24
Vitamin B12	4.70 (0.41)	1.89 (1.63)	0.19 (0.71)	0.07 (0.13)	−1.82 (0.30), 0.03 ^§^	−3.98 (0.60), 0.02 ^§^	−0.90 (0.44), 0.29
Vitamin C	42.88 (1.04)	56.50 (6.41)	32.18 (10.22)	47.04 (6.85)	0.45 (3.36), 0.91	2.69 (7.11), 0.74	−4.62 (10.97), 0.75
Vitamin D	95.89 (0.31)	95.08 (3.49)	91.81 (11.34)	73.01 (7.38)	−5.69 (1.99), 0.10	−9.54 (7.10), 0.31	−11.08 (4.35), 0.24
Vitamin E	84.38 (0.85)	87.14 (5.76)	90.63 (4.46)	82.39 (3.90)	0.57 (1.26), 0.70	2.19 (2.43), 0.46	−2.41 (3.28), 0.60
Dietary Fiber ^1^	12.74 (0.65)	2.80 (1.60)	6.67 (2.70)	12.01 (4.17)	−1.64 (1.86), 0.47	−5.51 (2.76), 0.18	4.61 (0.41), 0.06
Protein	1.92 (0.21)	1.36 (1.41)	0.27 (0.45)	0.14 (0.32)	−0.65 (0.07), 0.013 ^§^	−1.32 (0.40), 0.08	−0.61 (0.27), 0.26

EAR, estimated average requirement; AI, adequate intake. ^1^ >AI for potassium and dietary fiber. ^2^ Quantile 1 represents non-consumers; the remaining sample of those reporting intake (consumers) was divided into tertiles (quantiles 2, 3, and 4). ^3^ From regression analysis among all individuals, test for trend. ^4^ From regression analysis among all individuals, test for differences between non-consumers (Q1) and consumers (Q2,3,4). ^5^ From regression analysis among consumers (Q2–Q4), test for trend. ^†^ Statistically significant at *p* < 0.01; ^§^ Statistically significant at *p* < 0.05.

**Table 7:** Trends in % <EAR (>AI ^1^) for selected nutrients by beverage source of added sugars among adults (71+ y, n = 5639) from the pooled sample (NHANES 2003–2018, n = 35,128); weighted n’s are shown in the table.

Sweetened Beverages							
	Q1 ^2^ n = 15,059,331 % <EAR (SE)	Q2 n = 4,368,470 % <EAR (SE)	Q3 n = 3,489,802 % <EAR (SE)	Q4 n = 1,968,156 % <EAR (SE)	Quantile Trend ^3^ Beta (SE), *p*	Q1 vs. Q2,3,4 ^4^ Beta (SE), *p*	Q2–4 Trend ^5^ Beta (SE), *p*
Calcium	71.08 (1.31)	69.14 (1.83)	75.08 (2.09)	73.95 (2.64)	1.20 (1.00), 0.35	1.17 (2.58), 0.69	2.84 (2.12), 0.41
Magnesium	63.48 (1.19)	66.70 (2.17)	71.61 (2.19)	74.92 (2.55)	3.88 (0.22), 0.00 ^†^	6.71 (3.08), 0.16	4.21 (0.48), 0.07
Potassium ^1^	32.45 (1.24)	27.65 (2.19)	22.42 (2.01)	23.16 (2.55)	−3.85 (0.81), 0.04 ^§^	−7.59 (2.30), 0.08	−2.62 (1.78), 0.38
Vitamin A	31.18 (1.50)	30.41 (2.27)	40.14 (2.64)	41.62 (3.10)	3.65 (1.33), 0.11	5.04 (4.79), 0.40	6.12 (2.47), 0.24
Vitamin B12	5.02 (0.73)	3.42 (0.93)	4.71 (1.27)	4.18 (1.08)	−0.29 (0.37), 0.52	−0.98 (0.54), 0.21	0.49 (0.55), 0.53
Vitamin C	40.36 (1.47)	30.91 (2.27)	37.30 (2.44)	31.76 (3.03)	−2.67 (2.01), 0.32	−7.05 (2.73), 0.12	1.17 (3.57), 0.80
Vitamin D	92.99 (0.76)	91.71 (1.11)	95.42 (0.91)	94.42 (1.15)	0.67 (0.64), 0.41	0.60 (1.58), 0.74	1.65 (1.41), 0.45
Vitamin E	87.98 (0.81)	87.84 (1.57)	91.92 (1.13)	94.08 (1.57)	1.92 (0.58), 0.08	2.64 (2.39), 0.38	3.24 (0.57), 0.11
Dietary Fiber ^1^	13.51 (0.78)	10.35 (1.32)	6.22 (0.97)	5.65 (1.08)	−3.01 (0.42), 0.02 ^§^	−5.62 (2.02), 0.11	−2.57 (1.07), 0.25
Protein	5.69 (0.60)	6.10 (1.25)	6.58 (0.95)	4.69 (1.31)	−0.03 (0.33), 0.94	0.27 (0.66), 0.72	−0.56 (0.71), 0.58
**Soft Drinks**							
	**Q1 ^2^** **n = 19,417,069** **% <EAR (SE)**	**Q2** **n = 2,365,757** **% <EAR (SE)**	**Q3** **n = 1,957,503** **% <EAR (SE)**	**Q4** **n = 1,145,430% <EAR (SE)**	**Quantile Trend ^3^** **Beta (SE), *p***	**Q1 vs. Q2,3,4 ^4^** **Beta (SE), *p***	**Q2–4 Trend ^5^** **Beta (SE), *p***
Calcium	69.00 (1.18)	76.51 (1.96)	81.17 (2.24)	80.07 (3.02)	4.92 (1.12), 0.05 ^§^	9.91 (1.76), 0.03 ^§^	2.19 (1.76), 0.43
Magnesium	61.85 (1.02)	77.72 (2.20)	81.14 (2.48)	81.06 (2.70)	8.42 (2.33), 0.07	17.79 (1.37), 0.01 ^†^	1.92 (1.07), 0.32
Potassium ^1^	32.82 (1.10)	19.18 (2.26)	16.73 (2.60)	18.76 (2.93)	−6.71 (2.23), 0.10	−14.61 (0.92), 0.00 ^†^	−0.53 (1.37), 0.76
Vitamin A	30.00 (1.24)	42.26 (2.93)	46.67 (4.05)	49.63 (4.61)	7.70 (1.39), 0.03 ^§^	15.35 (2.39), 0.02 ^§^	3.79 (0.44), 0.07
Vitamin B12	4.76 (0.65)	3.55 (1.01)	5.80 (1.51)	5.43 (2.03)	0.22 (0.41), 0.65	−0.02 (0.86), 0.99	1.12 (0.80), 0.39
Vitamin C	34.49 (1.27)	44.16 (2.99)	52.34 (3.68)	43.49 (4.48)	5.82 (2.40), 0.14	12.48 (3.30), 0.06	0.87 (5.20), 0.89
Vitamin D	92.09 (0.65)	95.00 (1.12)	97.39 (0.76)	96.02 (1.07)	1.96 (0.55), 0.07	3.99 (0.87), 0.04 ^§^	0.78 (1.15), 0.62
Vitamin E	86.62 (0.81)	95.03 (1.14)	96.88 (0.88)	96.52 (1.35)	4.42 (1.26), 0.07	9.38 (0.71), 0.01 ^†^	0.90 (0.68), 0.41
Dietary Fiber ^1^	13.57 (0.70)	5.75 (1.04)	3.40 (0.81)	3.55 (1.10)	−4.38 (1.12), 0.06	−9.12 (0.93), 0.01 ^§^	−1.27 (0.76), 0.34
Protein	5.74 (0.57)	5.69 (1.18)	6.90 (1.26)	5.70 (1.57)	0.21 (0.23), 0.46	0.38 (0.47), 0.50	0.18 (0.74), 0.85
**Fruit Drinks**							
	**Q1 ^2^** **n = 20,768,028** **% <EAR (SE)**	**Q2** **n = 1,708,930** **% <EAR (SE)**	**Q3** **n = 1,541,335** **% <EAR (SE)**	**Q4** **n = 867,465** **% <EAR (SE)**	**Quantile Trend ^3^** **Beta (SE), *p***	**Q1 vs. Q2,3,4 ^4^** **Beta (SE), *p***	**Q2–4 Trend ^5^** **Beta (SE), *p***
Calcium	71.22 (1.06)	68.38 (3.02)	74.85 (3.10)	66.46 (4.28)	−0.43 (1.37), 0.78	−0.89 (2.76), 0.78	−0.15 (4.55), 0.98
Magnesium	65.42 (0.93)	62.05 (3.01)	69.47 (3.59)	65.72 (3.99)	0.53 (1.21), 0.70	0.20 (2.54), 0.94	2.44 (3.42), 0.61
Potassium ^1^	29.40 (1.05)	33.33 (3.33)	23.86 (3.48)	33.82 (3.65)	0.10 (1.81), 0.96	0.56 (3.63), 0.89	−0.81 (5.95), 0.91
Vitamin A	33.33 (1.33)	25.38 (2.83)	34.43 (3.95)	35.88 (3.82)	0.02 (2.00), 0.99	−2.23 (3.70), 0.61	5.66 (2.33), 0.25
Vitamin B12	4.75 (0.62)	2.43 (1.22)	5.13 (1.74)	2.32 (1.29)	−0.57 (0.57), 0.43	−1.36 (1.03), 0.32	0.24 (1.69), 0.91
Vitamin C	42.39 (1.21)	14.90 (3.11)	4.65 (3.87)	1.54 (1.37)	−16.69 (3.26), 0.04 ^§^	−34.30 (4.47), 0.02 ^§^	−7.06 (2.19), 0.19
Vitamin D	93.21 (0.54)	93.15 (1.63)	93.52 (1.49)	92.22 (2.54)	−0.13 (0.17), 0.53	−0.14 (0.38), 0.75	−0.38 (0.51), 0.60
Vitamin E	88.74 (0.65)	89.06 (2.20)	94.42 (1.94)	90.74 (2.36)	1.44 (0.83), 0.23	2.67 (1.86), 0.29	1.33 (2.77), 0.72
Dietary Fiber ^1^	11.49 (0.57)	13.56 (1.90)	6.64 (1.67)	8.50 (1.93)	−1.27 (0.99), 0.33	−1.62 (2.44), 0.57	−3.01 (2.69), 0.46
Protein	5.48 (0.47)	4.69 (1.68)	8.05 (2.15)	4.31 (1.46)	0.20 (0.65), 0.79	0.36 (1.32), 0.81	0.19 (2.18), 0.94
**Sports and Energy Drinks**							
	**Q1 ^2^** **n = 24,399,415** **% <EAR (SE)**	**Q2** **n = 230,617** **% <EAR (SE)**	**Q3** **n = 192,969** **% <EAR (SE)**	**Q4** **n = 132,236** **% <EAR (SE)**	**Quantile Trend ^3^** **Beta (SE), *p***	**Q1 vs. Q2,3,4 ^4^** **Beta (SE), *p***	**Q2–4 Trend ^5^** **Beta (SE), *p***
Calcium	71.32 (0.96)	70.47 (8.48)	46.56 (12.07)	75.64 (8.58)	−3.01 (4.45), 0.57	−6.92 (8.69), 0.51	0.72 (14.93), 0.97
Magnesium	65.70 (0.91)	57.64 (7.44)	44.56 (14.31)	67.07 (6.99)	−3.76 (3.67), 0.41	−9.60 (6.17), 0.26	3.46 (10.03), 0.79
Potassium ^1^	29.43 (0.96)	43.37 (9.51)	46.18 (10.97)	31.06 (7.04)	4.28 (3.41), 0.34	11.50 (4.35), 0.12	−5.52 (5.05), 0.47
Vitamin A	32.89 (1.20)	30.40 (8.98)	29.01 (11.18)	50.04 (8.50)	2.56 (2.73), 0.45	2.36 (6.41), 0.75	9.03 (6.32), 0.39
Vitamin B12	4.51 (0.53)	0.00 (0.68)	2.68 (4.57)	0.43 (1.33)	−1.54 (0.73), 0.17	−3.56 (0.84), 0.05	0.39 (1.39), 0.83
Vitamin C	37.09 (1.12)	39.71 (13.07)	29.32 (10.17)	39.76 (14.35)	−0.39 (1.69), 0.84	−0.61 (3.44), 0.88	−0.71 (5.87), 0.92
Vitamin D	93.30 (0.49)	89.88 (7.63)	91.06 (3.60)	95.12 (2.58)	−0.33 (0.93), 0.76	−1.65 (1.54), 0.40	2.52 (0.81), 0.20
Vitamin E	89.41 (0.63)	85.16 (5.54)	58.87 (19.32)	90.67 (5.06)	−4.83 (5.01), 0.44	−10.97 (9.53), 0.37	0.70 (16.36), 0.97
Dietary Fiber ^1^	11.27 (0.56)	11.89 (6.51)	20.27 (9.08)	10.70 (4.94)	1.32 (1.51), 0.47	2.91 (2.94), 0.43	0.03 (5.05), 1.00
Protein	5.59 (0.47)	4.01 (4.65)	2.18 (3.40)	10.39 (4.72)	0.27 (1.14), 0.83	−0.43 (2.33), 0.87	2.83 (2.83), 0.50
**Coffee and Tea**							
	**Q1 ^2^** **n = 21,570,292** **% <EAR (SE)**	**Q2** **n = 1,358,056** **% <EAR (SE)**	**Q3** **n = 1,043,372** **% <EAR (SE)**	**Q4** **n = 914,039** **% <EAR (SE)**	**Quantile Trend ^3^** **Beta (SE), *p***	**Q1 vs. Q2,3,4 ^4^** **Beta (SE), *p***	**Q2–4 Trend ^5^** **Beta (SE), *p***
Calcium	70.42 (1.00)	78.43 (2.84)	71.42 (3.30)	74.94 (5.01)	1.91 (1.75), 0.39	5.12 (2.30), 0.16	−2.40 (2.98), 0.57
Magnesium	64.81 (0.95)	71.02 (3.19)	65.15 (4.42)	68.79 (5.97)	1.49 (1.39), 0.39	3.94 (1.92), 0.18	−1.71 (2.69), 0.64
Potassium ^1^	29.82 (1.12)	23.50 (3.24)	28.82 (3.85)	29.93 (5.84)	−0.86 (1.54), 0.63	−3.29 (2.21), 0.27	3.48 (1.19), 0.21
Vitamin A	31.96 (1.18)	39.53 (4.33)	33.23 (4.78)	44.00 (7.01)	3.30 (1.67), 0.19	6.64 (2.99), 0.16	1.18 (4.83), 0.85
Vitamin B12	4.55 (0.56)	5.50 (1.91)	3.02 (1.76)	4.17 (2.45)	−0.22 (0.40), 0.64	−0.09 (0.82), 0.92	−0.89 (1.03), 0.55
Vitamin C	36.24 (1.16)	40.56 (4.31)	39.35 (3.97)	51.36 (7.25)	3.81 (1.19), 0.09	6.32 (3.56), 0.22	4.58 (3.74), 0.44
Vitamin D	92.72 (0.52)	96.87 (1.04)	91.97 (2.20)	97.02 (1.82)	1.14 (1.05), 0.39	2.70 (1.73), 0.26	−0.54 (2.81), 0.88
Vitamin E	88.83 (0.72)	93.05 (1.55)	88.25 (2.94)	92.27 (3.48)	1.02 (1.03), 0.43	2.59 (1.60), 0.25	−0.94 (2.49), 0.77
Dietary Fiber ^1^	11.75 (0.66)	9.64 (2.04)	9.12 (2.22)	5.23 (2.68)	−1.88 (0.30), 0.02 ^§^	−3.23 (1.33), 0.14	−2.00 (0.95), 0.28
Protein	5.34 (0.49)	8.54 (1.90)	4.34 (1.81)	6.47 (1.95)	0.40 (0.84), 0.68	1.47 (1.38), 0.40	−1.43 (1.79), 0.57
**Flavored Milk**							
	**Q1 ^2^** **n = 23,803,445** **% <EAR (SE)**	**Q2** **n = 375,093** **% <EAR (SE)**	**Q3** **n = 382,403** **% <EAR (SE)**	**Q4** **n = 324,817** **% <EAR (SE)**	**Quantile Trend ^3^** **Beta (SE), *p***	**Q1 vs. Q2,3,4 ^4^** **Beta (SE), *p***	**Q2–4 Trend ^5^** **Beta (SE), *p***
Calcium	72.01 (0.97)	61.22 (7.24)	58.26 (7.57)	30.89 (7.78)	−11.49 (2.20), 0.03 ^§^	−21.05 (9.70), 0.16	−14.80 (6.94), 0.28
Magnesium	65.75 (0.91)	71.19 (7.36)	52.40 (8.96)	36.71 (6.14)	−7.61 (2.98), 0.13	−11.22 (10.28), 0.39	−17.29 (0.88), 0.03 ^§^
Potassium ^1^	29.14 (0.95)	21.30 (7.46)	32.28 (8.56)	54.15 (6.44)	5.09 (3.53), 0.29	5.79 (9.83), 0.62	16.26 (3.10), 0.12
Vitamin A	33.12 (1.20)	28.84 (8.47)	23.18 (9.00)	19.99 (6.45)	−4.54 (0.20), 0.00 ^†^	−8.83 (2.68), 0.08	−4.46 (0.70), 0.10
Vitamin B12	4.62 (0.54)	8.62 (5.31)	0.22 (0.80)	0.34 (0.40)	−1.22 (1.16), 0.40	−1.25 (2.91), 0.71	−4.27 (2.42), 0.33
Vitamin C	37.11 (1.16)	35.64 (7.88)	30.16 (7.69)	37.72 (6.25)	−1.00 (1.19), 0.49	−2.60 (2.26), 0.37	0.84 (3.71), 0.86
Vitamin D	93.74 (0.48)	93.49 (3.64)	97.99 (3.60)	75.30 (8.07)	−3.28 (2.73), 0.35	−4.38 (6.90), 0.59	−8.69 (7.73), 0.46
Vitamin E	89.47 (0.65)	92.07 (2.72)	93.30 (3.95)	76.92 (7.28)	−1.88 (2.18), 0.48	−1.66 (5.26), 0.78	−7.31 (5.01), 0.38
Dietary Fiber ^1^	11.39 (0.57)	5.53 (2.97)	7.15 (3.34)	13.58 (5.77)	−0.62 (1.45), 0.71	−2.87 (2.48), 0.37	3.95 (1.37), 0.21
Protein	5.62 (0.45)	9.58 (4.66)	2.71 (1.68)	0.73 (1.00)	−1.14 (1.10), 0.41	−0.98 (2.79), 0.76	−4.50 (1.39), 0.19

EAR, estimated average requirement; AI, adequate intake. ^1^ >AI for potassium and dietary fiber. ^2^ Quantile 1 represents non-consumers; the remaining sample of those reporting intake (consumers) was divided into tertiles (quantiles 2, 3, and 4). ^3^ From regression analysis among all individuals, test for trend. ^4^ From regression analysis among all individuals, test for differences between non-consumers (Q1) and consumers (Q2,3,4). ^5^ From regression analysis among consumers (Q2-Q4), test for trend. ^†^ Statistically significant at *p* < 0.01; ^§^ Statistically significant at *p* < 0.05.

## Data Availability

The data used in this manuscript are publicly available at the NHANES website: https://wwwn.cdc.gov/nchs/nhanes/ (accessed on 26 July 2023).

## Supplementary Materials

The following supporting information can be downloaded at: https://www.mdpi.com/article/10.3390/nu15183916/s1, Figure S1: NHANES 2003-2018 analytic sample flow chart; Figure S2: Change in % <EAR (>AI ^†^) in association with increased added sugars intake from select beverage sources: A. soft drinks and B. fruit drinks, by NHANES 4-y cycles, among adults (19+ y); Change in % <EAR (>AI ^†^) in association with increased added sugars intake from select beverage sources: C. sports and energy drinks and D. coffee and tea, by NHANES 4-y cycles, among adults (19+ y); Table S1: Demographic description of adults (19+ y) in added sugars analyses from NHANES 2003–2018; Table S2: Percent of adults (19+ y) reporting intake (consumers) of beverage sources of added sugars from the pooled sample (NHANES 2003–2018, n = 35,128); Table S3: Trends in added sugars intake (% kcal) from beverage sources and the rest of the diet among all adults and consumers only from the pooled sample (NHANES 2003–2018, n = 35,128); Table S4: Trends in added sugars intake (grams) from beverage sources and the rest of the diet among all adults and consumers only from the pooled sample (NHANES 2003–2018, n = 35,128); Table S5: Association between added sugars intake from select beverages and % <EAR (>AI ^1^) among adults (19+ y) by NHANES 4-y cycles; Table S6: Quartiles of added sugars intake (% kcal) from the rest of the diet and trends in % <EAR (>AI ^1^) for selected nutrients among adults from the pooled sample (NHANES 2003–2018, n = 35,128); weighted n’s are shown in the table.

## Abbreviations

AI, Adequate Intake; DGA, Dietary Guidelines for Americans; EAR, Estimated Average Requirement; FPED, Food Patterns Equivalent Database; NCI, National Cancer Institute; UI, usual intake; WWEIA, What We Eat in America.
