# Supplementary material for: Intakes of Added Sugars, with a Focus on Beverages and the Associations with Nutrient Adequacy in US Adults (NHANES 2003–2018)
Source: Nutrients. 2023 Sep 9;15(18):3916. doi: 10.3390/nu15183916 (PMC10537713; doi:10.3390/nu15183916)
Supplement: Supplementary file 1 [file nutrients-15-03916-s001.zip › nutrients-2566061-supplementary.pdf]

**Supplemental Figure S1.** NHANES 2003-2018 analytic sample flow chart

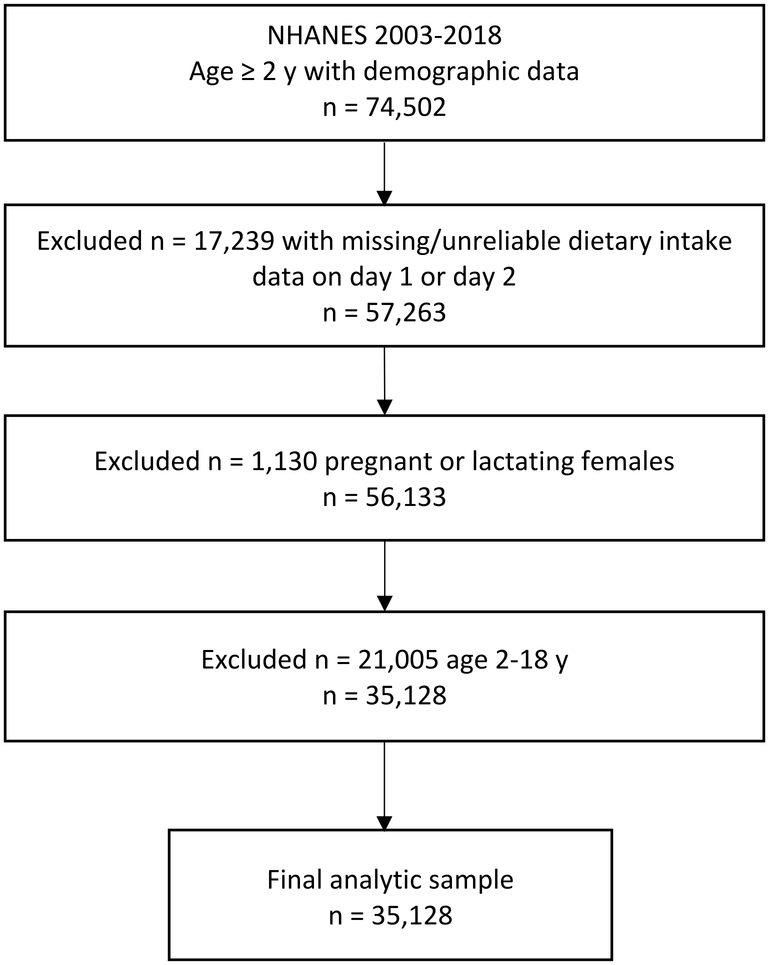

**Supplemental Table S1.** Demographic description of adults (19+ y) in added sugars analyses from NHANES 2003-2018

| Variable | Mean ± Standard Error |
|---|---|
| Age (mean) | 47.25 ± 0.23 |
| Gender = Male (%) | 48.76 ± 0.36 |
| Underweight[1] (%) | 1.54 ± 0.10 |
| Normal weight (%) | 29.01 ± 0.49 |
| Overweight (%) | 32.63 ± 0.47 |
| Obese (%) | 36.82 ± 0.56 |
| Race/Ethnicity = Mexican American (%) | 8.40 ± 0.63 |
| Race/Ethnicity = Other Hispanic (%) | 5.06 ± 0.39 |
| Race/Ethnicity = White (%) | 68.05 ± 1.21 |
| Race/Ethnicity = Black (%) | 11.40 ± 0.67 |
| Race/Ethnicity = Other (%) | 7.09 ± 0.35 |
| Poverty Income Ratio (PIR)[2] < 1.35 | 22.28 ± 0.66 |
| 1.35 <= PIR <= 1.85 | 9.87 ± 0.30 |
| PIR > 1.85 | 67.85 ± 0.80 |
| Physical Activity (PA)[3] = Sedentary | 24.05 ± 0.49 |
| PA = Moderate | 36.01 ± 0.47 |
| PA = Vigorous | 39.94 ± 0.59 |
| Education < HS (%) | 15.40 ± 0.51 |
| Education = HS/GED (%) | 23.89 ± 0.50 |
| Education = Some College (%) | 31.90 ± 0.49 |
| Education >/= Bachelor's Degree (%) | 28.80 ± 0.83 |
| Smoking = Current (%) | 19.24 ± 0.51 |
| Sample N | 35,128 |
| Population N | 175,304,193 |

[1]Underweight defined as a body mass index (BMI) < 18.5; normal weight defined as BMI of 18.5-24.9; overweight defined as BMI of 25-29.9; obesity defined as BMI ≥ 30

[2]Poverty income ratio based on household income compared to respective poverty level in the US

[3]Physical activity derived from responses to the physical activity questionnaire

**Supplemental Table S2.** Percent of adults (19+ y) reporting intake (consumers) of beverage sources of added sugars from the pooled sample (NHANES 2003-2018, n=35,128)

| Food Group | Sweetened Beverages | | Soft Drinks | | Fruit Drinks | | Sport and Energy Drinks | | Coffee and Tea | | Flavored Milk | |
|---|---|---|---|---|---|---|---|---|---|---|---|---|
| **19-50 y (n=18,110)** | | | | | | | | | | | | |
| **Year** | **Mean ± SE** | **P** | **Mean ± SE** | **P** | **Mean ± SE** | **P** | **Mean ± SE** | **P** | **Mean ± SE** | **P** | **Mean ± SE** | **P** |
| **2003-04** | 75.65 ± 1.32 | | 64.37 ± 1.46 | | 32.14 ± 2.19 | | 1.20 ± 0.26 | | 17.78 ± 1.98 | | 6.05 ± 0.80 | |
| **2005-06** | 64.32 ± 1.77 | <0.01* | 49.94 ± 1.95 | <0.01* | 25.80 ± 1.53 | 0.02 | 8.72 ± 0.78 | <0.01* | 18.08 ± 1.01 | 0.89 | 6.69 ± 0.67 | 0.54 |
| **2007-08** | 67.31 ± 1.90 | <0.01* | 52.89 ± 2.26 | <0.01* | 25.09 ± 2.13 | 0.02 | 8.56 ± 1.45 | <0.01* | 21.39 ± 1.87 | 0.19 | 4.57 ± 0.78 | 0.19 |
| **2009-10** | 63.72 ± 1.56 | <0.01* | 48.28 ± 1.22 | <0.01* | 25.65 ± 1.30 | 0.01 | 10.17 ± 0.59 | <0.01* | 19.32 ± 1.93 | 0.58 | 4.77 ± 0.47 | 0.17 |
| **2011-12** | 62.45 ± 2.48 | <0.01* | 46.27 ± 2.26 | <0.01* | 23.54 ± 2.44 | <0.01* | 12.95 ± 1.90 | <0.01* | 21.83 ± 1.34 | 0.09 | 4.50 ± 0.74 | 0.16 |
| **2013-14** | 62.30 ± 2.15 | <0.01* | 47.77 ± 1.93 | <0.01* | 18.40 ± 1.28 | <0.01* | 10.47 ± 0.63 | <0.01* | 22.31 ± 1.74 | 0.09 | 5.12 ± 0.53 | 0.34 |
| **2015-16** | 57.51 ± 2.43 | <0.01* | 42.49 ± 2.53 | <0.01* | 17.64 ± 1.05 | <0.01* | 9.88 ± 0.88 | <0.01* | 22.77 ± 2.06 | 0.08 | 3.95 ± 0.65 | 0.04 |
| **2017-18** | 56.53 ± 2.18 | <0.01* | 39.13 ± 2.81 | <0.01* | 17.29 ± 1.32 | <0.01* | 12.37 ± 1.33 | <0.01* | 27.81 ± 1.29 | <0.01* | 3.23 ± 0.52 | <0.01* |
| **Trend**[1] | **Beta ± SE** | **P** | **Beta ± SE** | **P** | **Beta ± SE** | **P** | **Beta ± SE** | **P** | **Beta ± SE** | **P** | **Beta ± SE** | **P** |
| | -2.19 ± 0.30 | <0.01 | -2.75 ± 0.35 | <0.01 | -1.98 ± 0.25 | <0.01 | 1.09 ± 0.15 | <0.01 | 1.18 ± 0.26 | <0.01 | -0.38 ± 0.10 | <0.01 |
| **51-70 y (n=11,379)** | | | | | | | | | | | | |
| **Year** | **Mean ± SE** | **P** | **Mean ± SE** | **P** | **Mean ± SE** | **P** | **Mean ± SE** | **P** | **Mean ± SE** | **P** | **Mean ± SE** | **P** |
| **2003-04** | 53.82 ± 2.70 | | 38.32 ± 2.62 | | 21.28 ± 1.38 | | 0.08 ± 0.06 | | 12.65 ± 1.80 | | 3.78 ± 0.88 | |
| **2005-06** | 49.73 ± 2.02 | 0.23 | 33.85 ± 2.11 | 0.19 | 20.02 ± 1.59 | 0.55 | 4.43 ± 0.87 | <0.01* | 12.95 ± 0.89 | 0.88 | 3.21 ± 0.49 | 0.57 |
| **2007-08** | 46.86 ± 2.47 | 0.06 | 32.77 ± 2.51 | 0.13 | 18.06 ± 1.20 | 0.08 | 2.65 ± 0.45 | <0.01* | 16.82 ± 1.65 | 0.09 | 3.85 ± 0.75 | 0.95 |
| **2009-10** | 42.92 ± 2.08 | <0.01* | 26.62 ± 1.79 | <0.01* | 20.76 ± 1.17 | 0.78 | 2.69 ± 0.34 | <0.01* | 18.07 ± 2.43 | 0.08 | 4.17 ± 0.84 | 0.75 |
| **2011-12** | 48.00 ± 2.36 | 0.11 | 33.14 ± 2.70 | 0.17 | 18.10 ± 1.81 | 0.16 | 3.94 ± 1.02 | <0.01* | 17.35 ± 2.26 | 0.11 | 3.16 ± 0.91 | 0.63 |
| **2013-14** | 45.50 ± 2.66 | 0.03 | 30.86 ± 2.30 | 0.03 | 12.46 ± 1.31 | <0.01* | 3.93 ± 0.70 | <0.01* | 16.03 ± 1.57 | 0.16 | 2.13 ± 0.39 | 0.09 |
| **2015-16** | 40.26 ± 2.29 | <0.01* | 29.00 ± 1.74 | <0.01* | 11.52 ± 1.64 | <0.01* | 4.47 ± 0.68 | <0.01* | 20.26 ± 2.60 | 0.02 | 4.08 ± 0.92 | 0.82 |
| **2017-18** | 44.07 ± 3.21 | 0.02 | 31.91 ± 3.47 | 0.14 | 13.92 ± 1.59 | <0.01* | 4.56 ± 0.98 | <0.01* | 18.11 ± 2.58 | 0.09 | 2.73 ± 0.50 | 0.30 |
| **Trend**[1] | **Beta ± SE** | **P** | **Beta ± SE** | **P** | **Beta ± SE** | **P** | **Beta ± SE** | **P** | **Beta ± SE** | **P** | **Beta ± SE** | **P** |
| | -1.31 ± 0.43 | <0.01 | -0.73 ± 0.43 | 0.09 | -1.36 ± 0.24 | <0.01 | 0.42 ± 0.12 | <0.01 | 0.82 ± 0.33 | 0.02 | -0.11 ± 0.11 | 0.29 |
| **71+ y (n=5,639)** | | | | | | | | | | | | |
| **Year** | **Mean ± SE** | **P** | **Mean ± SE** | **P** | **Mean ± SE** | **P** | **Mean ± SE** | **P** | **Mean ± SE** | **P** | **Mean ± SE** | **P** |
| **2003-04** | 42.36 ± 2.33 | | 23.88 ± 2.32 | | 21.09 ± 2.11 | | 0.00 ± 0.00 | | 12.56 ± 3.13 | | 4.48 ± 1.04 | |
| **2005-06** | 42.08 ± 3.09 | 0.94 | 24.70 ± 2.66 | 0.82 | 18.74 ± 1.48 | 0.36 | 1.90 ± 0.60 | <0.01* | 9.13 ± 1.40 | 0.32 | 5.62 ± 0.81 | 0.39 |
| **2007-08** | 43.05 ± 2.40 | 0.84 | 23.24 ± 1.87 | 0.83 | 21.12 ± 2.62 | 0.99 | 1.55 ± 0.76 | 0.04 | 12.83 ± 1.25 | 0.94 | 4.73 ± 0.96 | 0.86 |
| **2009-10** | 37.99 ± 1.88 | 0.15 | 22.08 ± 2.03 | 0.56 | 17.90 ± 1.79 | 0.25 | 1.38 ± 0.42 | <0.01* | 12.81 ± 1.58 | 0.95 | 3.51 ± 0.95 | 0.49 |
| **2011-12** | 39.18 ± 2.03 | 0.31 | 21.08 ± 1.96 | 0.36 | 17.06 ± 1.79 | 0.15 | 4.29 ± 1.30 | <0.01* | 10.91 ± 1.65 | 0.64 | 5.03 ± 1.18 | 0.73 |
| **2013-14** | 39.01 ± 3.18 | 0.40 | 19.86 ± 2.29 | 0.22 | 17.29 ± 2.01 | 0.19 | 0.96 ± 0.41 | 0.02 | 14.63 ± 2.53 | 0.61 | 2.85 ± 0.91 | 0.24 |
| **2015-16** | 38.53 ± 2.24 | 0.24 | 20.95 ± 2.27 | 0.37 | 11.20 ± 1.31 | <0.01* | 1.99 ± 0.75 | <0.01* | 16.40 ± 2.81 | 0.36 | 4.91 ± 1.11 | 0.78 |
| **2017-18** | 35.06 ± 2.71 | 0.04 | 20.72 ± 2.31 | 0.34 | 10.81 ± 1.46 | <0.01* | 3.26 ± 0.69 | <0.01* | 15.78 ± 2.04 | 0.39 | 3.72 ± 0.86 | 0.58 |
| **Trend**[1] | **Beta ± SE** | **P** | **Beta ± SE** | **P** | **Beta ± SE** | **P** | **Beta ± SE** | **P** | **Beta ± SE** | **P** | **Beta ± SE** | **P** |
| | -0.96 ± 0.40 | 0.02 | -0.60 ± 0.36 | 0.10 | -1.50 ± 0.27 | <0.01 | 0.29 ± 0.09 | <0.01 | 0.76 ± 0.37 | 0.04 | -0.14 ± 0.15 | 0.34 |

*Significantly different from reference cycle (2003-04), p<0.01

[1]Linear trend across all cycles, statistically significant at p<0.01

**Supplemental Table S3.** Trends in added sugars intake (% kcal) from beverage sources and the rest of the diet among all adults and consumers only from the pooled sample (NHANES 2003-2018, n=35,128)

| Food Group | Sweetened Beverages | | Soft Drinks | | Fruit Drinks | | Sport and Energy Drinks | | Coffee and Tea | | Flavored Milk | | Rest of the Diet | |
|---|---|---|---|---|---|---|---|---|---|---|---|---|---|---|
| | | | | | | | **All Individuals 19-50 y** | | | | | | | |
| **Year** | **Mean ± SE** | **P** | **Mean ± SE** | **P** | **Mean ± SE** | **P** | **Mean ± SE** | **P** | **Mean ± SE** | **P** | **Mean ± SE** | **P** | **Mean ± SE** | **P** |
| **2003-04** | 8.46 ± 0.32 | | 6.63 ± 0.35 | | 1.73 ± 0.16 | | 0.05 ± 0.02 | | 0.69 ± 0.10 | | 0.20 ± 0.04 | | 7.04 ± 0.18 | |
| **2005-06** | 6.75 ± 0.37 | <0.01* | 4.92 ± 0.30 | <0.01* | 1.42 ± 0.12 | 0.13 | 0.33 ± 0.04 | <0.01* | 0.82 ± 0.10 | 0.39 | 0.15 ± 0.02 | 0.27 | 7.14 ± 0.19 | 0.72 |
| **2007-08** | 7.12 ± 0.61 | 0.05 | 5.27 ± 0.58 | 0.05 | 1.35 ± 0.12 | 0.06 | 0.43 ± 0.07 | <0.01* | 0.94 ± 0.08 | 0.07 | 0.11 ± 0.02 | 0.05 | 7.15 ± 0.14 | 0.64 |
| **2009-10** | 6.29 ± 0.24 | <0.01* | 4.43 ± 0.21 | <0.01* | 1.38 ± 0.08 | 0.05 | 0.43 ± 0.04 | <0.01* | 0.97 ± 0.14 | 0.11 | 0.10 ± 0.01 | 0.01 | 7.10 ± 0.11 | 0.78 |
| **2011-12** | 5.73 ± 0.31 | <0.01* | 3.90 ± 0.30 | <0.01* | 1.22 ± 0.16 | 0.02 | 0.59 ± 0.09 | <0.01* | 1.26 ± 0.11 | <0.01* | 0.09 ± 0.02 | 0.02 | 6.79 ± 0.18 | 0.32 |
| **2013-14** | 5.82 ± 0.32 | <0.01* | 4.39 ± 0.27 | <0.01* | 0.77 ± 0.06 | <0.01* | 0.57 ± 0.06 | <0.01* | 1.25 ± 0.16 | <0.01* | 0.11 ± 0.01 | 0.03 | 6.82 ± 0.20 | 0.40 |
| **2015-16** | 4.85 ± 0.32 | <0.01* | 3.56 ± 0.28 | <0.01* | 0.67 ± 0.05 | <0.01* | 0.54 ± 0.08 | <0.01* | 1.29 ± 0.14 | <0.01* | 0.09 ± 0.01 | <0.01* | 6.46 ± 0.19 | 0.03 |
| **2017-18** | 4.60 ± 0.36 | <0.01* | 3.19 ± 0.32 | <0.01* | 0.65 ± 0.06 | <0.01* | 0.68 ± 0.10 | <0.01* | 1.32 ± 0.10 | <0.01* | 0.08 ± 0.02 | <0.01* | 6.82 ± 0.23 | 0.45 |
| **Trend[1]** | **Beta ± SE** | **P** | **Beta ± SE** | **P** | **Beta ± SE** | **P** | **Beta ± SE** | **P** | **Beta ± SE** | **P** | **Beta ± SE** | **P** | **Beta ± SE** | **P** |
| | -0.49 ± 0.06 | <0.01 | -0.40 ± 0.05 | <0.01 | -0.16 ± 0.02 | <0.01 | 0.07 ± 0.01 | <0.01 | 0.09 ± 0.02 | <0.01 | -0.01 ± 0.00 | <0.01 | -0.07 ± 0.03 | 0.01 |
| | | | | | | | **All Individuals 51-70 y** | | | | | | | |
| **Year** | **Mean ± SE** | **P** | **Mean ± SE** | **P** | **Mean ± SE** | **P** | **Mean ± SE** | **P** | **Mean ± SE** | **P** | **Mean ± SE** | **P** | **Mean ± SE** | **P** |
| **2003-04** | 4.24 ± 0.37 | | 3.06 ± 0.35 | | 1.09 ± 0.17 | | 0.00 ± 0.00 | | 0.51 ± 0.07 | | 0.08 ± 0.02 | | 8.08 ± 0.18 | |
| **2005-06** | 3.86 ± 0.27 | 0.41 | 2.79 ± 0.21 | 0.50 | 0.84 ± 0.10 | 0.21 | 0.18 ± 0.04 | <0.01* | 0.66 ± 0.08 | 0.17 | 0.07 ± 0.01 | 0.52 | 7.71 ± 0.21 | 0.18 |
| **2007-08** | 3.44 ± 0.30 | 0.10 | 2.54 ± 0.25 | 0.23 | 0.70 ± 0.10 | 0.05 | 0.12 ± 0.03 | <0.01* | 0.64 ± 0.08 | 0.19 | 0.07 ± 0.01 | 0.73 | 8.20 ± 0.16 | 0.62 |
| **2009-10** | 3.03 ± 0.21 | <0.01* | 1.81 ± 0.18 | <0.01* | 1.03 ± 0.10 | 0.75 | 0.12 ± 0.02 | <0.01* | 0.94 ± 0.18 | 0.02 | 0.12 ± 0.03 | 0.33 | 7.95 ± 0.16 | 0.59 |
| **2011-12** | 3.43 ± 0.22 | 0.06 | 2.28 ± 0.23 | 0.06 | 0.90 ± 0.13 | 0.38 | 0.22 ± 0.06 | <0.01* | 0.96 ± 0.18 | 0.02 | 0.05 ± 0.02 | 0.33 | 7.76 ± 0.21 | 0.25 |
| **2013-14** | 3.18 ± 0.27 | 0.02 | 2.31 ± 0.24 | 0.08 | 0.55 ± 0.06 | <0.01* | 0.16 ± 0.04 | <0.01* | 0.81 ± 0.10 | 0.01 | 0.08 ± 0.02 | 0.88 | 7.86 ± 0.22 | 0.42 |
| **2015-16** | 3.21 ± 0.36 | 0.05 | 2.52 ± 0.30 | 0.24 | 0.50 ± 0.09 | <0.01* | 0.13 ± 0.02 | <0.01* | 1.46 ± 0.32 | <0.01* | 0.09 ± 0.02 | 0.81 | 7.86 ± 0.17 | 0.36 |
| **2017-18** | 3.24 ± 0.46 | 0.09 | 2.48 ± 0.43 | 0.29 | 0.53 ± 0.07 | <0.01* | 0.18 ± 0.04 | <0.01* | 1.25 ± 0.32 | 0.02 | 0.08 ± 0.02 | 0.84 | 7.92 ± 0.17 | 0.50 |
| **Trend[1]** | **Beta ± SE** | **P** | **Beta ± SE** | **P** | **Beta ± SE** | **P** | **Beta ± SE** | **P** | **Beta ± SE** | **P** | **Beta ± SE** | **P** | **Beta ± SE** | **P** |
| | -0.12 ± 0.06 | 0.05 | -0.06 ± 0.05 | 0.29 | -0.07 ± 0.02 | <0.01 | 0.01 ± 0.00 | <0.01 | 0.12 ± 0.04 | <0.01 | 0.00 ± 0.00 | 0.95 | -0.02 ± 0.03 | 0.51 |
| | | | | | | | **All Individuals 71+ y** | | | | | | | |
| **Year** | **Mean ± SE** | **P** | **Mean ± SE** | **P** | **Mean ± SE** | **P** | **Mean ± SE** | **P** | **Mean ± SE** | **P** | **Mean ± SE** | **P** | **Mean ± SE** | **P** |
| **2003-04** | 2.57 ± 0.20 | | 1.60 ± 0.18 | | 0.79 ± 0.08 | | 0.00 ± 0.00 | | 0.70 ± 0.27 | | 0.12 ± 0.03 | | 9.22 ± 0.20 | |
| **2005-06** | 2.16 ± 0.20 | 0.15 | 1.43 ± 0.19 | 0.52 | 0.63 ± 0.06 | 0.12 | 0.05 ± 0.02 | 0.03 | 0.45 ± 0.15 | 0.42 | 0.14 ± 0.02 | 0.56 | 9.19 ± 0.25 | 0.92 |
| **2007-08** | 2.36 ± 0.13 | 0.36 | 1.37 ± 0.13 | 0.31 | 0.78 ± 0.10 | 0.92 | 0.11 ± 0.07 | 0.11 | 0.44 ± 0.05 | 0.35 | 0.12 ± 0.03 | 0.88 | 8.78 ± 0.15 | 0.09 |

| | | | | | | | | | | | | | | |
|---|---|---|---|---|---|---|---|---|---|---|---|---|---|---|
| **2009-10** | 2.40 ± 0.17 | 0.49 | 1.43 ± 0.16 | 0.48 | 0.88 ± 0.16 | 0.62 | 0.03 ± 0.01 | <0.01* | 0.47 ± 0.08 | 0.41 | 0.11 ± 0.03 | 0.83 | 8.66 ± 0.27 | 0.10 |
| **2011-12** | 2.03 ± 0.17 | 0.04 | 1.07 ± 0.13 | 0.02 | 0.75 ± 0.12 | 0.77 | 0.13 ± 0.05 | <0.01* | 0.58 ± 0.11 | 0.69 | 0.13 ± 0.03 | 0.82 | 9.41 ± 0.38 | 0.65 |
| **2013-14** | 2.26 ± 0.33 | 0.41 | 1.31 ± 0.26 | 0.36 | 0.73 ± 0.13 | 0.71 | 0.04 ± 0.02 | 0.05 | 1.05 ± 0.49 | 0.53 | 0.07 ± 0.02 | 0.20 | 8.73 ± 0.35 | 0.23 |
| **2015-16** | 2.00 ± 0.16 | 0.02 | 1.39 ± 0.17 | 0.41 | 0.40 ± 0.06 | <0.01* | 0.05 ± 0.02 | <0.01* | 0.92 ± 0.19 | 0.51 | 0.10 ± 0.03 | 0.65 | 8.26 ± 0.22 | <0.01* |
| **2017-18** | 2.10 ± 0.22 | 0.11 | 1.45 ± 0.18 | 0.55 | 0.44 ± 0.09 | <0.01* | 0.08 ± 0.02 | <0.01* | 0.76 ± 0.14 | 0.83 | 0.12 ± 0.03 | 0.98 | 8.56 ± 0.21 | 0.02 |
| **Trend**[1] | **Beta ± SE** | **P** | **Beta ± SE** | **P** | **Beta ± SE** | **P** | **Beta ± SE** | **P** | **Beta ± SE** | **P** | **Beta ± SE** | **P** | **Beta ± SE** | **P** |
| | -0.06 ± 0.03 | 0.07 | -0.02 ± 0.03 | 0.52 | -0.05 ± 0.01 | <0.01 | 0.00 ± 0.00 | 0.21 | 0.06 ± 0.03 | 0.08 | 0.00 ± 0.00 | 0.38 | -0.11 ± 0.03 | <0.01 |
| **Consumers Only 19-50 y** | | | | | | | | | | | | | | |
| **Year** | **Mean ± SE** | **P** | **Mean ± SE** | **P** | **Mean ± SE** | **P** | **Mean ± SE** | **P** | **Mean ± SE** | **P** | **Mean ± SE** | **P** | **Mean ± SE** | **P** |
| **2003-04** | 11.18 ± 0.37 | | 10.30 ± 0.45 | | 5.37 ± 0.21 | | 4.23 ± 1.87 | | 3.90 ± 0.27 | | 3.31 ± 0.36 | | 7.07 ± 0.18 | |
| **2005-06** | 10.50 ± 0.32 | 0.16 | 9.84 ± 0.31 | 0.41 | 5.51 ± 0.30 | 0.69 | 3.77 ± 0.27 | 0.80 | 4.54 ± 0.45 | 0.23 | 2.25 ± 0.24 | 0.01 | 7.16 ± 0.19 | 0.71 |
| **2007-08** | 10.58 ± 0.74 | 0.47 | 9.96 ± 0.76 | 0.70 | 5.38 ± 0.27 | 0.97 | 4.99 ± 0.65 | 0.70 | 4.40 ± 0.32 | 0.24 | 2.39 ± 0.27 | 0.04 | 7.20 ± 0.13 | 0.54 |
| **2009-10** | 9.88 ± 0.38 | 0.01 | 9.17 ± 0.37 | 0.06 | 5.37 ± 0.27 | 0.99 | 4.26 ± 0.30 | 0.99 | 5.04 ± 0.33 | <0.01* | 1.99 ± 0.13 | <0.01* | 7.13 ± 0.11 | 0.76 |
| **2011-12** | 9.18 ± 0.25 | <0.01* | 8.43 ± 0.33 | <0.01* | 5.19 ± 0.31 | 0.63 | 4.52 ± 0.24 | 0.88 | 5.78 ± 0.47 | <0.01* | 2.10 ± 0.17 | <0.01* | 6.82 ± 0.17 | 0.32 |
| **2013-14** | 9.34 ± 0.32 | <0.01* | 9.18 ± 0.31 | 0.04 | 4.17 ± 0.16 | <0.01* | 5.42 ± 0.49 | 0.54 | 5.59 ± 0.50 | <0.01* | 2.20 ± 0.14 | <0.01* | 6.84 ± 0.19 | 0.40 |
| **2015-16** | 8.43 ± 0.44 | <0.01* | 8.37 ± 0.54 | <0.01* | 3.79 ± 0.20 | <0.01* | 5.48 ± 0.47 | 0.52 | 5.68 ± 0.32 | <0.01* | 2.24 ± 0.27 | 0.02 | 6.51 ± 0.19 | 0.04 |
| **2017-18** | 8.13 ± 0.39 | <0.01* | 8.15 ± 0.42 | <0.01* | 3.76 ± 0.34 | <0.01* | 5.49 ± 0.64 | 0.53 | 4.74 ± 0.29 | 0.04 | 2.57 ± 0.40 | 0.17 | 6.89 ± 0.22 | 0.54 |
| **Trend**[1] | **Beta ± SE** | **P** | **Beta ± SE** | **P** | **Beta ± SE** | **P** | **Beta ± SE** | **P** | **Beta ± SE** | **P** | **Beta ± SE** | **P** | **Beta ± SE** | **P** |
| | -0.43 ± 0.06 | <0.01 | -0.31 ± 0.07 | <0.01 | -0.26 ± 0.04 | <0.01 | 0.25 ± 0.10 | 0.01 | 0.16 ± 0.05 | <0.01 | -0.09 ± 0.05 | 0.11 | -0.07 ± 0.03 | 0.02 |
| **Consumers Only 51-70 y** | | | | | | | | | | | | | | |
| **Year** | **Mean ± SE** | **P** | **Mean ± SE** | **P** | **Mean ± SE** | **P** | **Mean ± SE** | **P** | **Mean ± SE** | **P** | **Mean ± SE** | **P** | **Mean ± SE** | **P** |
| **2003-04** | 7.87 ± 0.44 | | 8.00 ± 0.51 | | 5.11 ± 0.62 | | 3.87 ± 1.27 | | 4.01 ± 0.35 | | 2.14 ± 0.30 | | 8.11 ± 0.18 | |
| **2005-06** | 7.76 ± 0.36 | 0.84 | 8.25 ± 0.41 | 0.70 | 4.21 ± 0.33 | 0.20 | 4.17 ± 0.56 | 0.83 | 5.06 ± 0.57 | 0.12 | 2.03 ± 0.19 | 0.75 | 7.72 ± 0.21 | 0.17 |
| **2007-08** | 7.33 ± 0.48 | 0.41 | 7.76 ± 0.53 | 0.75 | 3.90 ± 0.39 | 0.10 | 4.47 ± 0.64 | 0.67 | 3.83 ± 0.31 | 0.71 | 1.87 ± 0.18 | 0.44 | 8.21 ± 0.16 | 0.68 |
| **2009-10** | 7.07 ± 0.31 | 0.14 | 6.79 ± 0.43 | 0.07 | 4.95 ± 0.31 | 0.82 | 4.36 ± 0.40 | 0.71 | 5.21 ± 0.45 | 0.04 | 2.87 ± 0.42 | 0.17 | 7.96 ± 0.16 | 0.53 |
| **2011-12** | 7.14 ± 0.28 | 0.16 | 6.87 ± 0.31 | 0.06 | 4.96 ± 0.49 | 0.86 | 5.69 ± 1.96 | 0.44 | 5.53 ± 0.84 | 0.10 | 1.65 ± 0.42 | 0.35 | 7.79 ± 0.21 | 0.25 |
| **2013-14** | 6.99 ± 0.36 | 0.12 | 7.47 ± 0.41 | 0.42 | 4.39 ± 0.36 | 0.32 | 4.19 ± 0.58 | 0.82 | 5.07 ± 0.55 | 0.10 | 3.59 ± 0.54 | 0.02 | 7.87 ± 0.22 | 0.41 |
| **2015-16** | 7.98 ± 0.75 | 0.90 | 8.69 ± 0.81 | 0.47 | 4.35 ± 0.43 | 0.32 | 2.84 ± 0.31 | 0.43 | 7.18 ± 0.82 | <0.01* | 2.17 ± 0.27 | 0.95 | 7.87 ± 0.17 | 0.34 |
| **2017-18** | 7.36 ± 0.69 | 0.53 | 7.77 ± 0.87 | 0.82 | 3.83 ± 0.23 | 0.06 | 3.92 ± 0.63 | 0.97 | 6.90 ± 1.06 | 0.01 | 2.76 ± 0.22 | 0.10 | 7.93 ± 0.17 | 0.46 |
| **Trend**[1] | **Beta ± SE** | **P** | **Beta ± SE** | **P** | **Beta ± SE** | **P** | **Beta ± SE** | **P** | **Beta ± SE** | **P** | **Beta ± SE** | **P** | **Beta ± SE** | **P** |
| | -0.04 ± 0.09 | 0.63 | 0.00 ± 0.11 | 0.98 | -0.08 ± 0.07 | 0.22 | -0.16 ± 0.11 | 0.16 | 0.45 ± 0.13 | <0.01 | 0.09 ± 0.05 | 0.06 | -0.02 ± 0.03 | 0.50 |
| **Consumers Only 71+ y** | | | | | | | | | | | | | | |
| **Year** | **Mean ± SE** | **P** | **Mean ± SE** | **P** | **Mean ± SE** | **P** | **Mean ± SE** | **P**[2] | **Mean ± SE** | **P** | **Mean ± SE** | **P** | **Mean ± SE** | **P** |

| | | | | | | | | | | | | | | | | |
|---|---|---|---|---|---|---|---|---|---|---|---|---|---|---|---|---|
| **2003-04** | 6.08 ± 0.36 | | 6.69 ± 0.47 | | 3.75 ± 0.35 | | NA | NA | 5.55 ± 1.00 | | 2.58 ± 0.32 | | 9.22 ± 0.20 | |
| **2005-06** | 5.14 ± 0.22 | 0.03 | 5.80 ± 0.37 | 0.14 | 3.34 ± 0.27 | 0.36 | 2.59 ± 0.90 | 0.87 | 4.92 ± 1.25 | 0.70 | 2.42 ± 0.22 | 0.67 | 9.19 ± 0.25 | 0.92 |
| **2007-08** | 5.47 ± 0.26 | 0.18 | 5.90 ± 0.43 | 0.21 | 3.68 ± 0.31 | 0.88 | 6.83 ± 3.21 | 0.18 | 3.41 ± 0.22 | 0.04 | 2.58 ± 0.24 | 0.99 | 8.79 ± 0.15 | 0.09 |
| **2009-10** | 6.30 ± 0.40 | 0.67 | 6.47 ± 0.47 | 0.73 | 4.91 ± 0.66 | 0.12 | 2.18 ± 0.48 | 0.81 | 3.64 ± 0.50 | 0.09 | 3.02 ± 0.40 | 0.39 | 8.67 ± 0.27 | 0.11 |
| **2011-12** | 5.19 ± 0.33 | 0.07 | 5.06 ± 0.35 | <0.01* | 4.38 ± 0.48 | 0.29 | 2.97 ± 0.43 | 0.50 | 5.32 ± 0.58 | 0.84 | 2.50 ± 0.16 | 0.81 | 9.44 ± 0.38 | 0.62 |
| **2013-14** | 5.79 ± 0.46 | 0.62 | 6.58 ± 0.60 | 0.88 | 4.24 ± 0.40 | 0.36 | 3.97 ± 1.22 | 0.27 | 7.18 ± 2.33 | 0.52 | 2.41 ± 0.33 | 0.71 | 8.74 ± 0.35 | 0.23 |
| **2015-16** | 5.19 ± 0.21 | 0.03 | 6.66 ± 0.55 | 0.96 | 3.56 ± 0.52 | 0.77 | 2.40 ± 0.58 | 1.00 | 5.58 ± 0.75 | 0.98 | 1.99 ± 0.21 | 0.12 | 8.29 ± 0.22 | <0.01* |
| **2017-18** | 6.00 ± 0.47 | 0.89 | 6.98 ± 0.46 | 0.66 | 4.04 ± 0.65 | 0.70 | 2.39 ± 0.73 | | 4.84 ± 0.39 | 0.51 | 3.14 ± 0.42 | 0.29 | 8.58 ± 0.21 | 0.03 |
| **Trend**[1] | **Beta ± SE** | **P** | **Beta ± SE** | **P** | **Beta ± SE** | **P** | **Beta ± SE** | **P** | **Beta ± SE** | **P** | **Beta ± SE** | **P** | **Beta ± SE** | **P** |
| | -0.01 ± 0.06 | 0.89 | 0.09 ± 0.07 | 0.23 | 0.06 ± 0.07 | 0.37 | -0.27 ± 0.24[2] | 0.27[2] | 0.13 ± 0.14 | 0.35 | 0.00 ± 0.05 | 0.97 | -0.10 ± 0.03 | <0.01 |

*Significantly different from reference cycle (2003-04), p<0.01

[1]Linear trend across all cycles, statistically significant at p<0.01

NA: Not available; there were no consumers of this beverage for this age group

[2]Given there were no consumers of this beverage for this age group, the trend Beta is for 2005-2018 and the p-value represents the comparison of all other values to values for 2017-18 rather than for 2003-04

**Supplemental Table S4.** Trends in added sugars intake (grams) from beverage sources and the rest of the diet among all adults and consumers only from the pooled sample (NHANES 2003-2018, n=35,128)

| Food Group | Sweetened Beverages | | Soft Drinks | | Fruit Drinks | | Sport and Energy Drinks | | Coffee and Tea | | Flavored Milk | | Rest of the Diet | |
|---|---|---|---|---|---|---|---|---|---|---|---|---|---|---|
| **All Individuals 19-50 y** | | | | | | | | | | | | | | |
| **Year** | **Mean ± SE** | **P** | **Mean ± SE** | **P** | **Mean ± SE** | **P** | **Mean ± SE** | **P** | **Mean ± SE** | **P** | **Mean ± SE** | **P** | **Mean ± SE** | **P** |
| **2003-04** | 51.11 ± 2.13 | | 39.90 ± 2.31 | | 10.69 ± 0.97 | | 0.25 ± 0.05 | | 3.96 ± 0.68 | | 1.14 ± 0.20 | | 41.77 ± 1.01 | |
| **2005-06** | 39.24 ± 1.70 | <0.01* | 29.06 ± 1.41 | <0.01* | 7.72 ± 0.67 | 0.01 | 2.08 ± 0.18 | <0.01* | 4.70 ± 0.59 | 0.42 | 0.95 ± 0.13 | 0.45 | 40.92 ± 1.39 | 0.62 |
| **2007-08** | 40.16 ± 2.98 | <0.01* | 29.93 ± 3.00 | <0.01* | 7.47 ± 0.68 | <0.01* | 2.45 ± 0.39 | <0.01* | 4.51 ± 0.46 | 0.50 | 0.55 ± 0.10 | 0.01 | 39.62 ± 1.18 | 0.17 |
| **2009-10** | 36.51 ± 1.77 | <0.01* | 25.82 ± 1.51 | <0.01* | 7.82 ± 0.53 | 0.01 | 2.55 ± 0.20 | <0.01* | 5.19 ± 0.65 | 0.19 | 0.54 ± 0.08 | <0.01* | 39.70 ± 0.99 | 0.15 |
| **2011-12** | 32.67 ± 1.77 | <0.01* | 22.04 ± 1.79 | <0.01* | 7.00 ± 0.87 | <0.01* | 3.50 ± 0.57 | <0.01* | 7.02 ± 0.71 | <0.01* | 0.54 ± 0.11 | 0.01 | 38.34 ± 1.16 | 0.03 |
| **2013-14** | 32.45 ± 2.24 | <0.01* | 24.71 ± 2.00 | <0.01* | 4.03 ± 0.30 | <0.01* | 3.23 ± 0.37 | <0.01* | 6.77 ± 0.80 | <0.01* | 0.62 ± 0.05 | 0.02 | 37.51 ± 1.36 | 0.01 |
| **2015-16** | 26.62 ± 2.01 | <0.01* | 19.73 ± 1.87 | <0.01* | 3.44 ± 0.23 | <0.01* | 2.97 ± 0.36 | <0.01* | 7.19 ± 0.74 | <0.01* | 0.50 ± 0.08 | <0.01* | 35.37 ± 1.11 | <0.01* |
| **2017-18** | 25.89 ± 1.89 | <0.01* | 18.34 ± 1.76 | <0.01* | 3.49 ± 0.25 | <0.01* | 3.70 ± 0.56 | <0.01* | 7.11 ± 0.62 | <0.01* | 0.47 ± 0.10 | <0.01* | 37.49 ± 1.27 | <0.01* |
| **Trend**[1] | **Beta ± SE** | **P** | **Beta ± SE** | **P** | **Beta ± SE** | **P** | **Beta ± SE** | **P** | **Beta ± SE** | **P** | **Beta ± SE** | **P** | **Beta ± SE** | **P** |
| | -3.17 ± 0.33 | <0.01 | -2.58 ± 0.32 | <0.01 | -0.99 ± 0.10 | <0.01 | 0.38 ± 0.06 | <0.01 | 0.51 ± 0.10 | <0.01 | -0.08 ± 0.02 | <0.01 | -0.78 ± 0.18 | <0.01 |
| **All Individuals 51-70 y** | | | | | | | | | | | | | | |
| **Year** | **Mean ± SE** | **P** | **Mean ± SE** | **P** | **Mean ± SE** | **P** | **Mean ± SE** | **P** | **Mean ± SE** | **P** | **Mean ± SE** | **P** | **Mean ± SE** | **P** |
| **2003-04** | 21.29 ± 2.05 | | 15.22 ± 1.73 | | 5.70 ± 1.13 | | 0.02 ± 0.01 | | 2.42 ± 0.32 | | 0.44 ± 0.12 | | 39.26 ± 1.39 | |
| **2005-06** | 20.11 ± 1.61 | 0.65 | 14.43 ± 1.33 | 0.72 | 4.34 ± 0.49 | 0.27 | 1.19 ± 0.24 | <0.01* | 3.17 ± 0.40 | 0.15 | 0.35 ± 0.05 | 0.48 | 39.38 ± 1.73 | 0.96 |
| **2007-08** | 17.24 ± 1.58 | 0.12 | 12.86 ± 1.30 | 0.28 | 3.50 ± 0.50 | 0.08 | 0.59 ± 0.13 | <0.01* | 3.39 ± 0.46 | 0.09 | 0.36 ± 0.08 | 0.60 | 41.31 ± 0.91 | 0.22 |
| **2009-10** | 15.46 ± 1.11 | 0.01 | 9.26 ± 0.92 | <0.01* | 5.25 ± 0.61 | 0.72 | 0.60 ± 0.08 | <0.01* | 4.69 ± 0.89 | 0.02 | 0.65 ± 0.16 | 0.27 | 41.80 ± 1.29 | 0.18 |
| **2011-12** | 17.83 ± 1.22 | 0.15 | 11.64 ± 1.20 | 0.09 | 4.79 ± 0.82 | 0.51 | 1.26 ± 0.37 | <0.01* | 5.23 ± 1.21 | 0.03 | 0.27 ± 0.08 | 0.24 | 40.37 ± 1.40 | 0.58 |
| **2013-14** | 16.53 ± 1.55 | 0.07 | 12.03 ± 1.43 | 0.16 | 2.95 ± 0.30 | 0.02 | 0.74 ± 0.15 | <0.01* | 4.23 ± 0.49 | <0.01* | 0.31 ± 0.07 | 0.33 | 40.27 ± 1.54 | 0.63 |
| **2015-16** | 17.06 ± 2.11 | 0.15 | 13.48 ± 1.85 | 0.49 | 2.55 ± 0.48 | 0.01 | 0.72 ± 0.14 | <0.01* | 7.74 ± 1.58 | <0.01* | 0.48 ± 0.11 | 0.80 | 39.89 ± 1.09 | 0.72 |
| **2017-18** | 17.33 ± 2.09 | 0.18 | 13.01 ± 1.98 | 0.40 | 2.90 ± 0.43 | 0.02 | 1.15 ± 0.34 | <0.01* | 6.28 ± 1.63 | 0.02 | 0.36 ± 0.07 | 0.58 | 42.44 ± 1.55 | 0.13 |
| **Trend**[1] | **Beta ± SE** | **P** | **Beta ± SE** | **P** | **Beta ± SE** | **P** | **Beta ± SE** | **P** | **Beta ± SE** | **P** | **Beta ± SE** | **P** | **Beta ± SE** | **P** |
| | -0.46 ± 0.30 | 0.13 | -0.19 ± 0.27 | 0.49 | -0.37 ± 0.11 | <0.01 | 0.08 ± 0.04 | 0.04 | 0.62 ± 0.18 | <0.01 | -0.01 ± 0.01 | 0.68 | 0.24 ± 0.22 | 0.29 |
| **All Individuals 71+ y** | | | | | | | | | | | | | | |
| **Year** | **Mean ± SE** | **P** | **Mean ± SE** | **P** | **Mean ± SE** | **P** | **Mean ± SE** | **P** | **Mean ± SE** | **P** | **Mean ± SE** | **P** | **Mean ± SE** | **P** |

| | | | | | | | | | | | | | | |
|---|---|---|---|---|---|---|---|---|---|---|---|---|---|---|
| **2003-04** | 10.28 ± 0.79 | | 6.15 ± 0.63 | | 3.27 ± 0.34 | | 0.00 ± 0.00 | | 2.38 ± 0.84 | | 0.53 ± 0.15 | | 38.89 ± 1.11 | |
| **2005-06** | 8.78 ± 0.85 | 0.20 | 5.79 ± 0.70 | 0.71 | 2.53 ± 0.33 | 0.12 | 0.21 ± 0.09 | 0.02 | 1.74 ± 0.43 | 0.50 | 0.58 ± 0.10 | 0.78 | 40.41 ± 1.64 | 0.44 |
| **2007-08** | 9.43 ± 0.58 | 0.39 | 5.48 ± 0.50 | 0.41 | 3.12 ± 0.38 | 0.78 | 0.41 ± 0.24 | 0.09 | 1.79 ± 0.22 | 0.50 | 0.46 ± 0.09 | 0.71 | 36.99 ± 1.33 | 0.28 |
| **2009-10** | 9.41 ± 0.63 | 0.39 | 5.94 ± 0.66 | 0.82 | 3.12 ± 0.46 | 0.80 | 0.13 ± 0.04 | <0.01* | 1.93 ± 0.34 | 0.62 | 0.42 ± 0.13 | 0.62 | 37.65 ± 1.59 | 0.52 |
| **2011-12** | 8.97 ± 0.64 | 0.20 | 4.56 ± 0.50 | 0.05 | 3.36 ± 0.54 | 0.88 | 0.59 ± 0.19 | <0.01* | 2.79 ± 0.53 | 0.68 | 0.59 ± 0.16 | 0.78 | 43.03 ± 2.55 | 0.14 |
| **2013-14** | 10.22 ± 1.69 | 0.98 | 6.28 ± 1.42 | 0.93 | 3.01 ± 0.55 | 0.69 | 0.16 ± 0.08 | 0.04 | 7.06 ± 4.28 | 0.28 | 0.33 ± 0.09 | 0.28 | 40.70 ± 2.11 | 0.45 |
| **2015-16** | 8.65 ± 0.61 | 0.11 | 5.75 ± 0.66 | 0.66 | 2.00 ± 0.39 | 0.02 | 0.27 ± 0.10 | <0.01* | 4.08 ± 0.69 | 0.12 | 0.44 ± 0.12 | 0.67 | 37.25 ± 1.43 | 0.37 |
| **2017-18** | 9.02 ± 0.85 | 0.28 | 6.07 ± 0.68 | 0.94 | 1.95 ± 0.38 | 0.01 | 0.36 ± 0.11 | <0.01* | 3.41 ± 0.58 | 0.31 | 0.50 ± 0.12 | 0.91 | 41.77 ± 1.08 | 0.07 |
| **Trend**[1] | **Beta ± SE** | **P** | **Beta ± SE** | **P** | **Beta ± SE** | **P** | **Beta ± SE** | **P** | **Beta ± SE** | **P** | **Beta ± SE** | **P** | **Beta ± SE** | **P** |
| | -0.09 ± 0.13 | 0.47 | 0.01 ± 0.11 | 0.93 | -0.15 ± 0.06 | <0.01 | 0.03 ± 0.02 | 0.07 | 0.41 ± 0.16 | 0.01 | -0.01 ± 0.02 | 0.55 | 0.24 ± 0.20 | 0.25 |
| **Consumers Only 19-50 y** | | | | | | | | | | | | | | |
| **Year** | **Mean ± SE** | **P** | **Mean ± SE** | **P** | **Mean ± SE** | **P** | **Mean ± SE** | **P** | **Mean ± SE** | **P** | **Mean ± SE** | **P** | **Mean ± SE** | **P** |
| **2003-04** | 67.57 ± 2.39 | | 61.99 ± 2.93 | | 33.27 ± 1.32 | | 21.09 ± 1.90 | | 22.27 ± 1.82 | | 18.78 ± 1.77 | | 41.90 ± 1.02 | |
| **2005-06** | 61.00 ± 1.40 | 0.02 | 58.19 ± 1.53 | 0.25 | 29.90 ± 1.34 | 0.08 | 23.89 ± 0.87 | 0.18 | 25.97 ± 2.43 | 0.22 | 14.22 ± 1.56 | 0.06 | 41.06 ± 1.37 | 0.62 |
| **2007-08** | 59.67 ± 3.56 | 0.07 | 56.60 ± 3.64 | 0.25 | 29.77 ± 1.35 | 0.07 | 28.64 ± 2.66 | 0.02 | 21.11 ± 1.30 | 0.60 | 11.94 ± 0.94 | <0.01* | 39.92 ± 1.14 | 0.19 |
| **2009-10** | 57.30 ± 2.69 | <0.01* | 53.48 ± 2.79 | 0.04 | 30.47 ± 1.67 | 0.19 | 25.10 ± 1.10 | 0.07 | 26.87 ± 1.47 | 0.05 | 11.24 ± 0.90 | <0.01* | 39.86 ± 0.98 | 0.15 |
| **2011-12** | 52.32 ± 1.64 | <0.01* | 47.63 ± 2.23 | <0.01* | 29.75 ± 1.90 | 0.13 | 27.04 ± 1.89 | 0.03 | 32.16 ± 2.59 | <0.01* | 11.94 ± 0.72 | <0.01* | 38.46 ± 1.14 | 0.03 |
| **2013-14** | 52.09 ± 2.33 | <0.01* | 51.73 ± 2.88 | 0.01 | 21.93 ± 0.76 | <0.01* | 30.89 ± 3.16 | <0.01* | 30.35 ± 2.26 | <0.01* | 12.15 ± 0.82 | <0.01* | 37.62 ± 1.37 | 0.01 |
| **2015-16** | 46.28 ± 3.01 | <0.01* | 46.43 ± 3.90 | <0.01* | 19.52 ± 0.92 | <0.01* | 30.03 ± 1.79 | <0.01* | 31.56 ± 1.95 | <0.01* | 12.71 ± 1.43 | <0.01* | 35.64 ± 1.12 | <0.01* |
| **2017-18** | 45.79 ± 2.08 | <0.01* | 46.87 ± 2.33 | <0.01* | 20.18 ± 1.84 | <0.01* | 29.90 ± 3.90 | 0.04 | 25.58 ± 1.63 | 0.18 | 14.48 ± 1.96 | 0.11 | 37.88 ± 1.26 | 0.01 |
| **Trend**[1] | **Beta ± SE** | **P** | **Beta ± SE** | **P** | **Beta ± SE** | **P** | **Beta ± SE** | **P** | **Beta ± SE** | **P** | **Beta ± SE** | **P** | **Beta ± SE** | **P** |
| | -3.05 ± 0.38 | <0.01 | -2.26 ± 0.46 | <0.01 | -1.92 ± 0.22 | <0.01 | 0.99 ± 0.52 | 0.06 | 0.90 ± 0.29 | <0.01 | -0.58 ± 0.28 | 0.04 | -0.76 ± 0.18 | <0.01 |
| **Consumers Only 51-70 y** | | | | | | | | | | | | | | |
| **Year** | **Mean ± SE** | **P** | **Mean ± SE** | **P** | **Mean ± SE** | **P** | **Mean ± SE** | **P** | **Mean ± SE** | **P** | **Mean ± SE** | **P** | **Mean ± SE** | **P** |
| **2003-04** | 39.55 ± 2.48 | | 39.72 ± 2.69 | | 26.81 ± 4.40 | | 19.05 ± 2.45 | | 19.10 ± 1.68 | | 11.56 ± 1.01 | | 39.40 ± 1.43 | |
| **2005-06** | 40.44 ± 2.23 | 0.79 | 42.63 ± 2.81 | 0.46 | 21.70 ± 1.56 | 0.28 | 26.80 ± 3.29 | 0.06 | 24.44 ± 2.90 | 0.11 | 10.80 ± 0.99 | 0.59 | 39.44 ± 1.73 | 0.98 |
| **2007-08** | 36.78 ± 2.59 | 0.44 | 39.23 ± 2.86 | 0.90 | 19.40 ± 2.17 | 0.13 | 22.21 ± 3.36 | 0.45 | 20.17 ± 2.03 | 0.69 | 9.44 ± 0.82 | 0.11 | 41.36 ± 0.91 | 0.25 |
| **2009-10** | 36.02 ± 1.64 | 0.24 | 34.80 ± 2.03 | 0.15 | 25.29 ± 2.19 | 0.76 | 22.41 ± 2.33 | 0.32 | 25.97 ± 2.20 | 0.01 | 15.66 ± 2.01 | 0.07 | 41.83 ± 1.29 | 0.21 |
| **2011-12** | 37.14 ± 1.69 | 0.42 | 35.11 ± 1.49 | 0.14 | 26.45 ± 3.13 | 0.95 | 32.06 ± 11.44 | 0.27 | 30.17 ± 6.09 | 0.08 | 8.51 ± 1.63 | 0.11 | 40.51 ± 1.38 | 0.58 |
| **2013-14** | 36.34 ± 2.02 | 0.32 | 38.99 ± 2.56 | 0.84 | 23.70 ± 2.17 | 0.53 | 18.82 ± 2.42 | 0.95 | 26.36 ± 2.56 | 0.02 | 14.38 ± 1.15 | 0.07 | 40.35 ± 1.54 | 0.65 |
| **2015-16** | 42.37 ± 4.76 | 0.60 | 46.48 ± 5.24 | 0.25 | 22.16 ± 1.81 | 0.33 | 16.05 ± 1.77 | 0.32 | 38.21 ± 4.06 | <0.01* | 11.72 ± 1.35 | 0.92 | 39.96 ± 1.10 | 0.75 |

| | | | | | | | | | | | | | | |
|---|---|---|---|---|---|---|---|---|---|---|---|---|---|---|
| **2017-18** | 39.33 ± 3.28 | 0.96 | 40.77 ± 3.88 | 0.82 | 20.81 ± 1.37 | 0.20 | 25.28 ± 6.08 | 0.34 | 34.67 ± 5.50 | <0.01* | 13.25 ± 1.22 | 0.29 | 42.49 ± 1.56 | 0.15 |
| **Trend**[1] | **Beta ± SE** | **P** | **Beta ± SE** | **P** | **Beta ± SE** | **P** | **Beta ± SE** | **P** | **Beta ± SE** | **P** | **Beta ± SE** | **P** | **Beta ± SE** | **P** |
| | 0.08 ± 0.48 | 0.86 | 0.31 ± 0.55 | 0.58 | -0.31 ± 0.44 | 0.48 | -0.78 ± 0.86 | 0.36 | 2.50 ± 0.66 | <0.01 | 0.24 ± 0.19 | 0.21 | 0.24 ± 0.23 | 0.30 |
| **Consumers Only<br>71+ y** | | | | | | | | | | | | | | |
| **Year** | **Mean ± SE** | **P** | **Mean ± SE** | **P** | **Mean ± SE** | **P** | **Mean ± SE** | **P**[2] | **Mean ± SE** | **P** | **Mean ± SE** | **P** | **Mean ± SE** | **P** |
| **2003-04** | 24.26 ± 1.30 | | 25.74 ± 1.47 | | 15.49 ± 1.38 | | NA | NA | 18.92 ± 2.92 | | 11.73 ± 1.74 | | 38.89 ± 1.11 | |
| **2005-06** | 20.85 ± 0.91 | 0.03 | 23.45 ± 1.39 | 0.26 | 13.51 ± 1.30 | 0.30 | 10.83 ± 3.48 | 0.94 | 19.10 ± 2.82 | 0.97 | 10.25 ± 1.19 | 0.48 | 40.41 ± 1.64 | 0.44 |
| **2007-08** | 21.91 ± 1.15 | 0.18 | 23.59 ± 1.47 | 0.30 | 14.78 ± 1.22 | 0.70 | 26.54 ± 11.45 | 0.20 | 13.91 ± 0.97 | 0.11 | 9.71 ± 0.65 | 0.28 | 37.00 ± 1.33 | 0.28 |
| **2009-10** | 24.77 ± 1.24 | 0.78 | 26.89 ± 1.83 | 0.62 | 17.42 ± 1.75 | 0.39 | 9.66 ± 1.92 | 0.69 | 15.06 ± 2.18 | 0.29 | 12.09 ± 1.58 | 0.88 | 37.70 ± 1.58 | 0.54 |
| **2011-12** | 22.89 ± 1.20 | 0.44 | 21.63 ± 1.09 | 0.03 | 19.69 ± 2.20 | 0.11 | 13.77 ± 1.92 | 0.49 | 25.60 ± 3.88 | 0.17 | 11.64 ± 1.17 | 0.97 | 43.13 ± 2.55 | 0.13 |
| **2013-14** | 26.20 ± 2.57 | 0.50 | 31.64 ± 3.81 | 0.15 | 17.41 ± 1.78 | 0.40 | 16.82 ± 4.49 | 0.31 | 48.26 ± 22.45 | 0.20 | 11.70 ± 1.69 | 0.99 | 40.72 ± 2.11 | 0.44 |
| **2015-16** | 22.44 ± 1.09 | 0.28 | 27.43 ± 2.41 | 0.55 | 17.88 ± 3.72 | 0.55 | 13.48 ± 1.32 | 0.51 | 24.91 ± 2.45 | 0.12 | 9.03 ± 1.17 | 0.20 | 37.40 ± 1.43 | 0.41 |
| **2017-18** | 25.72 ± 1.83 | 0.52 | 29.30 ± 1.72 | 0.12 | 18.04 ± 2.51 | 0.37 | 11.17 ± 3.26 | | 21.61 ± 1.68 | 0.43 | 13.50 ± 1.50 | 0.44 | 41.89 ± 1.09 | 0.06 |
| **Trend**[1] | **Beta ± SE** | **P** | **Beta ± SE** | **P** | **Beta ± SE** | **P** | **Beta ± SE** | **P** | **Beta ± SE** | **P** | **Beta ± SE** | **P** | **Beta ± SE** | **P** |
| | 0.33 ± 0.23 | 0.15 | 0.75 ± 0.29 | 0.01 | 0.62 ± 0.33 | 0.06 | -0.71 ± 0.93[2] | 0.44[2] | 1.66 ± 0.75 | 0.03 | 0.10 ± 0.23 | 0.65 | 0.26 ± 0.20 | 0.21 |

*Significantly different from reference cycle (2003-04), $p<0.01$

[1]Linear trend across all cycles, statistically significant at $p<0.01$

NA: Not available; there were no consumers of this beverage for this age group

[2]Given there were no consumers of this beverage for this age group, the trend Beta is for 2005-2018 and the p-value represents the comparison of all other values to values for 2017-18 rather than for 2003-04

**Supplemental Figure S2.** Change in % <EAR (>AI†) in association with increased added sugars intake from select beverage sources: **A.** soft drinks and **B.** fruit drinks, by NHANES 4-y cycles, among adults (19+ y)

**A. Soft Drinks**

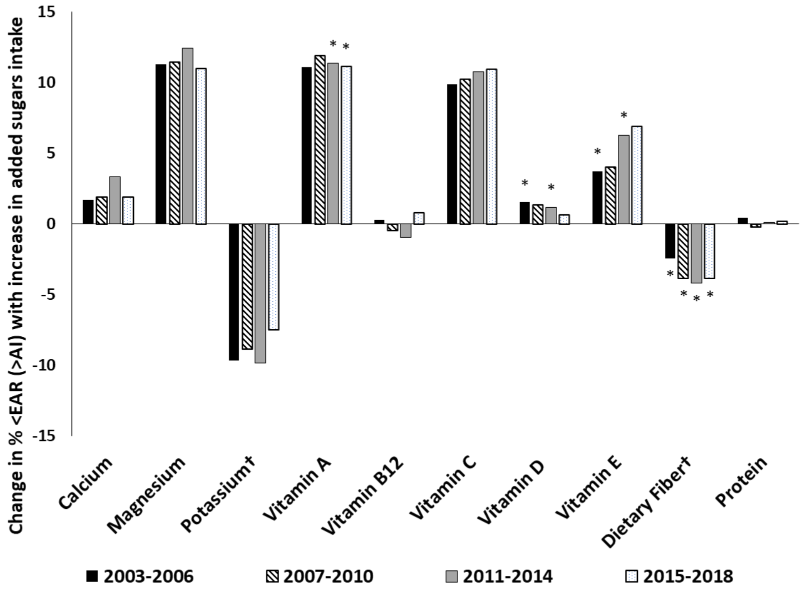


**B. Fruit Drinks**

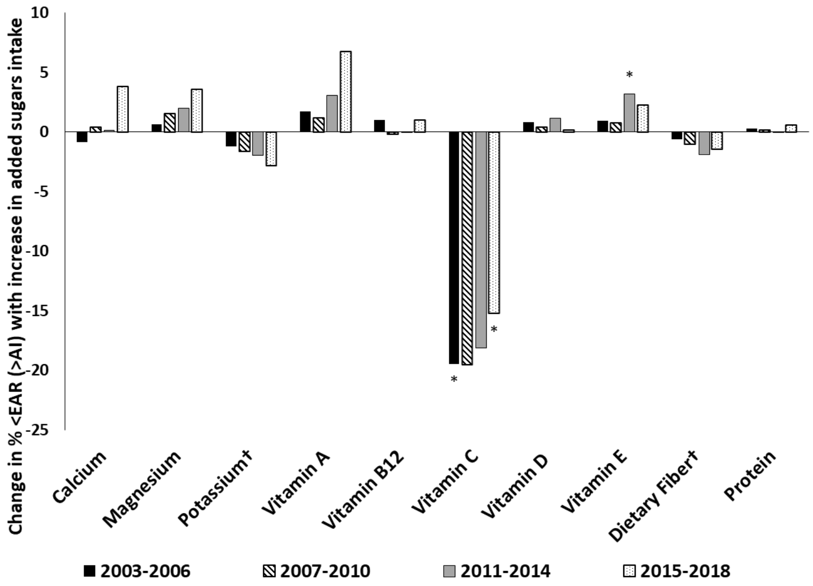


EAR, estimated average requirement; AI, adequate intake

†>AI for potassium and dietary fiber

*Statistically significant at $p<0.05$; from regression analysis, test for trend across quantiles of added sugars intake

Intakes of added sugars, with a focus on beverages and the associations with nutrient adequacy in US adults (NHANES 2003-2018), Ricciuto L et al.

**Supplemental Figure S2.** Change in % <EAR (>AI[†]) in association with increased added sugars intake from select beverage sources: **C.** sport and energy drinks and **D.** coffee and tea, by NHANES 4-y cycles, among adults (19+ y)

**C. Sport and Energy Drinks**

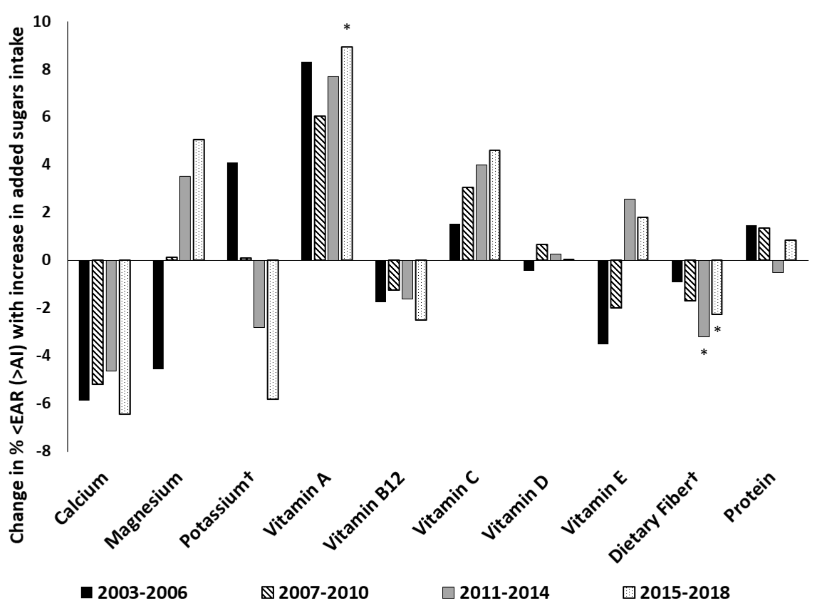


**1D Coffee and Tea**

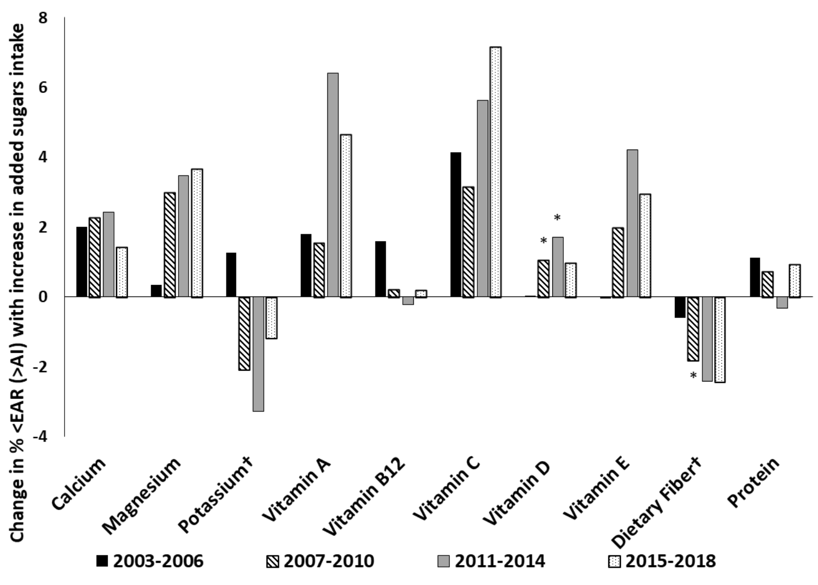


EAR, estimated average requirement; AI, adequate intake

†>AI for potassium and dietary fiber

*Statistically significant at $p<0.05$; from regression analysis, test for trend across quantiles of added sugars intake

**Supplemental Table S5.** Association between added sugars intake from select beverages and % <EAR (>AI[1]) among adults (19+ y) by NHANES 4-y cycles

| | **NHANES 2003-2006** | **NHANES 2007-2010** | **NHANES 2011-2014** | **NHANES 2015-2018** |
|---|---|---|---|---|
| | **Soft Drinks** | | | |
| **Nutrient** | **Beta (SE), P[2]** | **Beta (SE), P** | **Beta (SE), P** | **Beta (SE), P** |
| **Calcium** | 1.70 (4.68), 0.75 | 1.89 (5.21), 0.75 | 3.37 (4.45), 0.53 | 1.91 (3.90), 0.67 |
| **Magnesium** | 11.29 (4.39), 0.12 | 11.44 (4.21), 0.11 | 12.42 (3.87), 0.08 | 11.01 (4.46), 0.13 |
| **Potassium[1]** | -9.64 (3.75), 0.12 | -8.87 (3.49), 0.13 | -9.83 (2.57), 0.06 | -7.50 (2.84), 0.12 |
| **Vitamin A** | 11.08 (3.21), 0.07 | 11.91 (2.84), 0.05 | 11.36 (1.10), 0.01* | 11.15 (2.01), 0.03* |
| **Vitamin B12** | 0.27 (1.27), 0.85 | -0.45 (0.56), 0.50 | -0.93 (0.78), 0.35 | 0.81 (1.49), 0.64 |
| **Vitamin C** | 9.88 (4.37), 0.15 | 10.25 (3.83), 0.12 | 10.77 (2.64), 0.06 | 10.94 (2.67), 0.05 |
| **Vitamin D** | 1.54 (0.28), 0.03* | 1.35 (0.93), 0.28 | 1.19 (0.16), 0.02* | 0.65 (0.78), 0.49 |
| **Vitamin E** | 3.72 (0.78), 0.04* | 4.02 (1.28), 0.09 | 6.30 (0.97), 0.02* | 6.93 (2.50), 0.11 |
| **Dietary Fiber[1]** | -2.44 (0.36), 0.02* | -3.83 (0.65), 0.03* | -4.20 (0.41), 0.01* | -3.86 (0.34), 0.01* |
| **Protein** | 0.44 (0.86), 0.66 | -0.21 (0.57), 0.75 | 0.09 (0.49), 0.87 | 0.22 (0.74), 0.79 |
| | **Fruit Drinks** | | | |
| **Nutrient** | **Beta (SE), P** | **Beta (SE), P** | **Beta (SE), P** | **Beta (SE), P** |
| **Calcium** | -0.81 (3.00), 0.81 | 0.42 (2.66), 0.89 | 0.13 (2.63), 0.97 | 3.84 (4.57), 0.49 |
| **Magnesium** | 0.66 (3.97), 0.88 | 1.58 (3.88), 0.72 | 1.98 (3.32), 0.61 | 3.62 (3.61), 0.42 |
| **Potassium[1]** | -1.19 (3.64), 0.77 | -1.61 (2.99), 0.64 | -1.97 (2.19), 0.46 | -2.79 (2.24), 0.34 |
| **Vitamin A** | 1.72 (4.82), 0.76 | 1.19 (3.27), 0.75 | 3.06 (2.40), 0.33 | 6.79 (4.87), 0.30 |
| **Vitamin B12** | 1.03 (1.50), 0.56 | -0.19 (0.35), 0.65 | -0.05 (0.66), 0.95 | 1.00 (0.54), 0.20 |
| **Vitamin C** | -19.45 (4.20), 0.04* | -19.49 (5.09), 0.06 | -18.08 (4.24), 0.05 | -15.22 (2.45), 0.02* |
| **Vitamin D** | 0.82 (1.20), 0.57 | 0.43 (1.15), 0.74 | 1.16 (0.35), 0.08 | 0.21 (0.21), 0.41 |
| **Vitamin E** | 0.93 (1.13), 0.50 | 0.80 (1.59), 0.67 | 3.18 (0.45), 0.02* | 2.29 (3.57), 0.59 |
| **Dietary Fiber[1]** | -0.60 (0.24), 0.13 | -1.03 (0.40), 0.13 | -1.91 (1.34), 0.29 | -1.41 (0.51), 0.11 |
| **Protein** | 0.27 (0.70), 0.74 | 0.20 (0.40), 0.66 | -0.02 (0.52), 0.98 | 0.62 (0.57), 0.39 |
| | **Sport and Energy Drinks** | | | |
| **Nutrient** | **Beta (SE), P** | **Beta (SE), P** | **Beta (SE), P** | **Beta (SE), P** |
| **Calcium** | -5.87 (7.90), 0.53 | -5.19 (6.12), 0.49 | -4.65 (4.67), 0.42 | -6.44 (3.47), 0.20 |
| **Magnesium** | -4.55 (7.76), 0.62 | 0.11 (6.65), 0.99 | 3.50 (6.34), 0.64 | 5.04 (5.59), 0.46 |
| **Potassium[1]** | 4.10 (6.63), 0.60 | 0.10 (6.84), 0.99 | -2.82 (4.38), 0.59 | -5.82 (2.45), 0.14 |
| **Vitamin A** | 8.30 (5.97), 0.30 | 6.02 (3.55), 0.23 | 7.69 (2.07), 0.07 | 8.92 (1.74), 0.04* |
| **Vitamin B12** | -1.76 (0.56), 0.09 | -1.26 (0.38), 0.08 | -1.63 (0.66), 0.13 | -2.51 (0.66), 0.06 |
| **Vitamin C** | 1.54 (5.79), 0.81 | 3.05 (3.72), 0.50 | 3.99 (1.33), 0.10 | 4.60 (1.35), 0.08 |
| **Vitamin D** | -0.45 (2.19), 0.86 | 0.66 (0.55), 0.35 | 0.26 (0.88), 0.80 | 0.04 (0.70), 0.96 |
| **Vitamin E** | -3.53 (3.91), 0.46 | -2.01 (3.22), 0.60 | 2.55 (3.83), 0.57 | 1.80 (4.36), 0.72 |
| **Dietary Fiber[1]** | -0.92 (0.31), 0.10 | -1.70 (0.41), 0.05 | -3.21 (0.22), 0.00* | -2.27 (0.46), 0.04* |
| **Protein** | 1.48 (1.22), 0.35 | 1.33 (0.83), 0.25 | -0.51 (0.25), 0.18 | 0.83 (1.07), 0.52 |
| | **Coffee and Tea** | | | |
| **Nutrient** | **Beta (SE), P** | **Beta (SE), P** | **Beta (SE), P** | **Beta (SE), P** |

| | | | | |
|---|---|---|---|---|
| **Calcium** | 2.01 (2.36), 0.48 | 2.26 (4.15), 0.64 | 2.42 (2.52), 0.44 | 1.43 (4.18), 0.76 |
| **Magnesium** | 0.33 (3.02), 0.92 | 2.98 (3.53), 0.49 | 3.47 (2.76), 0.34 | 3.66 (5.00), 0.54 |
| **Potassium**[1] | 1.26 (2.90), 0.71 | -2.08 (2.51), 0.49 | -3.28 (3.00), 0.39 | -1.19 (3.34), 0.76 |
| **Vitamin A** | 1.80 (3.59), 0.67 | 1.55 (3.42), 0.69 | 6.40 (2.73), 0.14 | 4.66 (2.68), 0.22 |
| **Vitamin B12** | 1.58 (1.30), 0.35 | 0.22 (0.74), 0.80 | -0.23 (1.13), 0.86 | 0.19 (0.74), 0.82 |
| **Vitamin C** | 4.14 (3.35), 0.34 | 3.16 (4.42), 0.55 | 5.63 (3.42), 0.24 | 7.15 (2.41), 0.10 |
| **Vitamin D** | 0.02 (1.51), 0.99 | 1.05 (0.19), 0.03* | 1.70 (0.18), 0.01* | 0.97 (0.33), 0.10 |
| **Vitamin E** | -0.05 (0.80), 0.96 | 1.98 (1.21), 0.24 | 4.21 (1.45), 0.10 | 2.95 (2.49), 0.36 |
| **Dietary Fiber**[1] | -0.60 (0.86), 0.56 | -1.81 (0.38), 0.04* | -2.42 (0.95), 0.13 | -2.44 (0.97), 0.13 |
| **Protein** | 1.11 (0.63), 0.22 | 0.73 (0.72), 0.42 | -0.33 (0.29), 0.37 | 0.94 (0.87), 0.39 |

EAR, estimated average requirement; AI, adequate intake

[1]>AI for potassium and dietary fiber

[2]Beta represents the regression coefficient (change) in percentage of the population <EAR (>AI) across quantiles of added sugars from the select beverage, testing hypothesis that regression coefficient (slope) equals 0

*Statistically significant at p<0.05

**Supplemental Table S6.** Quartiles of added sugars intake (% kcal) from the rest of the diet and trends in % <EAR (>AI[1]) for selected nutrients among adults from the pooled sample (NHANES 2003-2018, n=35,128); weighted n's are shown in the table

| **19-50 y (n=18,110)** | | | | | |
|---|---|---|---|---|---|
| **Added Sugars** (% kcal) | **Q1** n=31,557,211 <3.48 | **Q2** n=31,579,320 3.48 to ≤6.08 | **Q3** n=31,574,933 >6.08 to ≤9.33 | **Q4** n=31,575,369 >9.33 | **Quartile Trend[2]** |
| | **% <EAR* (SE)** | **% <EAR* (SE)** | **% <EAR* (SE)** | **% <EAR* (SE)** | **Beta (SE), P** |
| **Calcium** | 54.98 (1.50) | 38.10 (1.32) | 26.05 (1.53) | 16.57 (0.93) | -12.80 (1.18), 0.01† |
| **Magnesium** | 69.85 (1.26) | 59.39 (1.54) | 49.00 (1.52) | 32.09 (1.33) | -12.30 (1.11), 0.01† |
| **Potassium[1]** | 16.02 (1.15) | 23.29 (1.30) | 31.59 (1.32) | 52.98 (1.42) | 11.78 (2.38), 0.04§ |
| **Vitamin A** | 70.25 (1.56) | 55.97 (1.68) | 43.59 (1.67) | 24.95 (1.65) | -14.78 (0.89), 0.00† |
| **Vitamin B12** | 10.63 (0.92) | 2.52 (0.48) | 1.69 (0.47) | 1.21 (0.32) | -2.98 (1.31), 0.15 |
| **Vitamin C** | 54.49 (1.42) | 51.08 (1.43) | 46.09 (1.73) | 41.28 (1.44) | -4.45 (0.26), 0.00† |
| **Vitamin D** | 98.89 (0.26) | 97.69 (0.45) | 95.06 (0.67) | 91.95 (0.87) | -2.32 (0.31), 0.02§ |
| **Vitamin E** | 93.69 (0.70) | 91.06 (1.28) | 84.77 (1.35) | 70.53 (1.48) | -7.46 (1.85), 0.06 |
| **Dietary Fiber[1]** | 1.79 (0.30) | 1.72 (0.30) | 2.03 (0.36) | 4.47 (0.62) | 0.81 (0.41), 0.19 |
| **Protein** | 4.92 (0.71) | 0.89 (0.20) | 0.16 (0.09) | 0.15 (0.07) | -1.54 (0.66), 0.15 |
| **51-70 y (n=11,379)** | | | | | |
| **Added Sugars** (% kcal) | **Q1** n=17,907,711 <4.41 | **Q2** n=17,073,548 4.41 to ≤7.21 | **Q3** n=17,258,187 >7.21 to ≤10.43 | **Q4** n=17,734,287 >10.43 | **Quartile Trend[2]** |
| | **% <EAR (SE)** | **% <EAR (SE)** | **% <EAR (SE)** | **% <EAR (SE)** | **Beta (SE), P** |
| **Calcium** | 68.78 (1.32) | 62.69 (1.65) | 48.62 (1.83) | 34.44 (1.60) | -11.49 (1.41), 0.01§ |
| **Magnesium** | 65.84 (1.52) | 63.59 (1.79) | 49.38 (2.20) | 33.10 (2.00) | -10.87 (2.34), 0.04§ |
| **Potassium[1]** | 24.78 (1.44) | 26.98 (1.57) | 39.48 (2.01) | 57.30 (2.06) | 10.61 (2.48), 0.05 |
| **Vitamin A** | 57.64 (1.77) | 48.74 (1.88) | 37.51 (2.40) | 21.72 (2.00) | -11.74 (1.06), 0.01† |
| **Vitamin B12** | 10.32 (1.05) | 3.38 (0.70) | 2.19 (0.64) | 1.13 (0.35) | -3.04 (1.02), 0.10 |
| **Vitamin C** | 45.97 (1.37) | 46.34 (2.27) | 41.81 (2.12) | 37.70 (2.03) | -2.81 (0.81), 0.07 |
| **Vitamin D** | 97.93 (0.38) | 97.45 (0.53) | 94.95 (0.62) | 92.18 (0.91) | -1.91 (0.39), 0.04§ |
| **Vitamin E** | 92.14 (0.93) | 91.54 (1.15) | 85.79 (1.75) | 71.12 (2.03) | -6.56 (2.17), 0.09 |
| **Dietary Fiber[1]** | 9.36 (0.92) | 8.26 (0.77) | 11.70 (1.23) | 18.43 (1.61) | 2.87 (1.23), 0.14 |
| **Protein** | 5.59 (0.67) | 1.57 (0.34) | 0.35 (0.12) | 0.28 (0.11) | -1.81 (0.63), 0.10 |
| **71+ y (n=5,639)** | | | | | |
| **Added Sugars** (% kcal) | **Q1** n=5,798,155 <5.27 | **Q2** n=6,615,333 5.27 to ≤8.33 | **Q3** n=6,471,226 >8.33 to ≤11.52 | **Q4** n=6,001,044 >11.52 | **Quartile Trend[2]** |
| | **% <EAR (SE)** | **% <EAR (SE)** | **% <EAR (SE)** | **% <EAR (SE)** | **Beta (SE), P** |
| **Calcium** | 85.95 (1.35) | 77.66 (1.98) | 67.60 (2.15) | 55.11 (1.83) | -10.21 (0.67), 0.00† |
| **Magnesium** | 76.67 (1.57) | 71.50 (1.61) | 64.67 (2.07) | 48.82 (1.95) | -8.91 (1.76), 0.04§ |
| **Potassium[1]** | 17.74 (1.47) | 22.76 (1.69) | 29.68 (2.12) | 46.59 (2.02) | 9.20 (1.95), 0.04§ |
| **Vitamin A** | 47.88 (2.35) | 36.73 (2.03) | 26.80 (2.28) | 14.73 (2.13) | -10.92 (0.28), 0.00† |

| | | | | | |
|---|---|---|---|---|---|
| **Vitamin B12** | 10.52 (1.73) | 3.06 (0.77) | 1.80 (0.63) | 1.55 (0.51) | -2.88 (1.24), 0.15 |
| **Vitamin C** | 43.28 (2.28) | 37.29 (2.17) | 34.52 (2.02) | 31.13 (1.79) | -3.94 (0.52), 0.02§ |
| **Vitamin D** | 96.25 (0.59) | 94.66 (0.79) | 93.01 (1.10) | 91.08 (1.00) | -1.71 (0.06), 0.00† |
| **Vitamin E** | 94.83 (0.83) | 93.98 (0.96) | 91.06 (1.19) | 80.93 (1.75) | -4.35 (1.51), 0.10 |
| **Dietary Fiber**[1] | 8.49 (0.92) | 7.69 (0.78) | 10.33 (1.17) | 15.47 (1.67) | 2.30 (0.96), 0.14 |
| **Protein** | 12.88 (1.49) | 5.04 (0.78) | 1.60 (0.42) | 1.38 (0.33) | -3.88 (1.23), 0.09 |

EAR, estimated average requirement; AI, adequate intake

[1]>AI for potassium and dietary fiber

[2]From regression analysis, test for trend

†Statistically significant at p<0.01; §Statistically significant at p<0.05
